# A systematic review of the efficacy of ketamine for craniofacial pain

**DOI:** 10.1080/24740527.2023.2210167

**Published:** 2023-06-26

**Authors:** Yasmine Hoydonckx, Tyler McKechnie, Miki Peer, Marina Englesakis, Pranab Kumar

**Affiliations:** a Department of Anesthesia and Pain Medicine, University of Toronto, Toronto Western Hospital, Toronto, Ontario, Canada; b Department of Surgery, Division of General Surgery, McMaster University, Hamilton, Ontario, Canada; c Department of Anesthesia and Pain Medicine, Toronto Western Hospital, Toronto, Ontario, Canada; d Library & Information Services, University Health Network, Toronto, Ontario, Canada

**Keywords:** craniofacial pain, headache, migraine, cluster headache, trigeminal neuralgia, trigeminal neuropathy, ketamine, chronic pain

## Abstract

**Background:**

Craniofacial pain (CFP) poses a burden on patients and health care systems. It is hypothesized that ketamine, an *N*-methyl-d-aspartate (NMDA) receptor antagonist, can reverse central sensitization associated with causation and propagation of CFP. This systematic review aims to assess the role of ketamine for CFP.

**Methods:**

Databases were searched for studies published up to September 26, 2022, investigating the efficacy of ketamine for adults with CFP. Primary outcome was the change in pain intensity at 60 min postintervention. Two reviewers screened and extracted data. Registration with PROSPERO was performed (CRD42020178649).

**Results:**

Twenty papers (six randomized controlled trials [RCTs], 14 observational studies) including 670 patients were identified. Substantial heterogeneity in terms of study design, population, dose, route of administration, treatment duration, and follow-up was noted. Bolus dose ranged from 0.2–0.3 mg/kg (intravenous) to 0.4 mg/kg (intramuscular) to 0.25–0.75 mg/kg (intranasal). Ketamine infusions (0.1–1 mg/kg/h) were given over various durations. Follow-up was short in RCTs (from 60 min to 72 h) but longer in observational studies (up to 18 months). Ketamine by bolus treatment failed to reduce migraine intensity but had an effect by reducing intensity of aura, cluster headache (CH), and trigeminal neuralgia. Prolonged ketamine infusions showed sustainable reduction of migraine intensity and frequency of CH attacks, but the quality of the evidence is low.

**Conclusion:**

Current evidence remains conflicting on the efficacy of ketamine for CFP owing to low quality and heterogeneity across studies. Ketamine infusions are suggested to provide sustained improvement, possibly because of prolonged duration and higher dosage of administration. RCTs should focus on the dose–response relationship of prolonged ketamine infusions on CFP.

## RÉSUMÉ

**Contexte:** La douleur crânio-faciale représente un fardeau pour les patients et les systèmes de soins de santé. L’hypothèse a été émise que la kétamine, un antagoniste du récepteur N-méthyl-D-aspartate (NMDA), peut inverser la sensibilisation centrale associée à la causalité et à la propagation de la douleur crânio-faciale. Cette revue systématique vise à évaluer le rôle de la kétamine dans la douleur crânio-faciale.

**Méthodes:** Les bases de données ont été consultées pour y repérer les études publiées jusqu’au 26 septembre 2022 qui portaient sur l’efficacité de la kétamine chez les adultes atteints de douleur crânio-faciale. Le critère de jugement principal était le changement de l’intensité de la douleur 60 minutes après l’intervention. Deux évaluateurs ont examiné et extrait les données. L’inscription auprès de PROSPERO a été réalisée (CRD42020178649).

**Résultats:** Vingt articles (six essais contrôlés randomisés, 14 études observationnelles) incluant 670 patients ont été répertoriées. Une hétérogénéité considérable en matière de devis d’étude, de population, de dose, de voie d’administration, de durée du traitement et de suivi a été notée. La dose bolus variait de 0,2 à 0,3 mg/kg (voie intraveineuse) à 0,4 mg/kg (voie intramusculaire) et à 0,25-0,75 mg/kg (voie intranasale). Les perfusions de kétamine (0,1-1 mg/kg/h) étaient administrées sur différentes durées. Le suivi était court dans les études contrôllées randomisées (de 60 min à 72 h) mais plus long dans les études observationnelles (jusqu’à 18 mois). La kétamine par traitement bolus n’a pas réussi à réduire l’intensité de la migraine mais a eu un effet en réduisant l’intensité de l’aura, la céphalée en grappe et la névralgie du trijumeau. Les perfusions de kétamine prolongées ont montré une réduction durable de l’intensité de la migraine et la fréquence des crises de CH, mais la qualité des données probantes est faible.

**Conclusions:** Les données probantes actuelles demeurent contradictoires sur l’efficacité de la kétamine pour la douleur crânio-faciale en raison de la faible qualité et de l’hétérogénéité des études. Il est suggéré que les perfusions de kétamine procurent une amélioration soutenue, peut-être en raison de leur durée prolongée et de leur posologie d’administration plus élevée. Les essais contrôlés randomisés devraient se concentrer sur la relation dose-réponse des perfusions prolongées de kétamine sur la douleur crâno-faciale.

## Introduction

### Description of the Condition

Craniofacial pain (CFP) is common, with a lifelong incidence of 96%, and poses a significant burden on patients’ quality of life and the health care system.^1–4^ Despite the availability of a variety of treatment modalities, many patients experience unsatisfactory pain relief and/or adverse effects from existing pharmacological interventions or cannot afford expensive treatments.^3,5^

It is hypothesized that CFP syndromes share a mechanism of central sensitization as a cause for pain. Central sensitization is characterized by an increase in neuronal excitability secondary to repetitive stimulation of the nociceptive C-fibers in the trigeminocervical complex and the brain, which is mediated by the activation of *N*-methyl-d-aspartate (NMDA) receptors.^6–8^ Activation of the NMDA receptors plays a major role in ongoing pain, opioid-induced hyperalgesia, and mood dysregulation, and it is considered the principal receptor involved in the phenomena of central sensitization and “wind-up,” resulting in hyperalgesia, allodynia, and spontaneous pain.^9,10^ This central sensitization can be reversed by blockade of these receptors by noncompetitive NMDA antagonists such as ketamine.^6,7,10,11^

Ketamine is a chemical derivative of phencyclidine with analgesic, dissociative, and psychomimetic properties.^12^ Its primary mechanism of action is as a noncompetitive antagonist of the NMDA receptors residing in the central nervous system.^10,13^ Ketamine is a versatile drug that can be administered via many routes, including intravenous (IV) and intramuscular (IM) but also oral, intranasal, inhalation, topical, and rectal, rendering it an easy agent for out-of-hospital care. It can be given as a single bolus or infusion or a combination of both. Although its potency is comparable to that of opioids, ketamine has a much better safety profile and is less likely to lead to development of tolerance. Possible adverse effects of ketamine include hypertension and tachycardia, hallucinations, and hepatic toxicity with chronic exposure.^14^

Ketamine has successfully been used in the treatment of complex chronic pain states such as complex regional pain syndrome and neuropathic pain,^11^ but its therapeutic role in CFP has not been completely established. Therefore, the objective of this systematic review was to evaluate the efficacy of ketamine for the treatment of CFP and examine its effects on pain-associated domains.

## Methods

### Registration

This systematic review was conducted according to the recommendations of the Cochrane Collaboration and was reported as per the Preferred Reporting Items for Systematic Reviews and Meta-Analysis (PRISMA) guidelines. The protocol of this systematic review was registered with PROSPERO (ID CRD42020178649).

### Data Sources and Search Strategy

We conducted a comprehensive search of the literature from inception to February 1, 2020. Updated searches were conducted over the same databases and clinical trial registries on November 3, 2020, and September 26, 2022, with the assistance of a medical information specialist (M.E.).

The following databases were searched: Embase (1947–), MEDLINE (1946–), MEDLINE ePubs, In-Process and Other Non-Indexed Citations, Cochrane Database of Systematic Reviews (2005–), Cochrane Central Register of Controlled Trials (1991–), and PubMed-NOT-MEDLINE. We also searched Web of Science Core Collection (1900–; Clarivate Analytics) and Scopus (1960–; Elsevier). Clinical trial registries, ClinicalTrials.Gov, and the World Health Organization International Clinical Trials Registry Platform were searched to identify trials.

We restricted our search to human subjects with moderate-to-severe pain. For Embase, MEDLINE, Cochrane CENTRAL, and Scopus, both controlled vocabulary terms (Embase-Emtree; MEDLINE-MeSH) and text word searching were conducted for each of the following segments: (“ketamine” or related synonyms) AND (“headache” or “migraine” or “cluster headache” or “facial pain” or “short-lasting unilateral neuralgiform headache attacks with conjunctival injection and tearing (SUNCT)” or “short-lasting unilateral neuralgiform headache attacks with cranial autonomic symptoms (SUNA)” or “trigeminal neuralgia” or “trigeminal autonomic cephalalgia (TAC)” or “occipital neuralgia” or “cephalalgia” or other related terms). The most recent Ovid Medline search strategy and the MEDLINE search strategy are provided in Appendix 1.

### Inclusion/Exclusion Criteria

The studies were screened for eligibility based on title, abstract, and subsequently full manuscript. Studies were included if they met the selection criteria as listed below.

#### Population

This review included studies of human subjects ≥18 years of age with a CFP (primary headache or neuropathic facial pain) diagnosis that fit the criteria of the *International Classification of Headache Disorders* third edition.^15^ Studies that investigated effects of ketamine on a mixed population of patients with pain for which data on CFP could not be extracted separately were excluded. No restrictions were put in terms of chronicity, frequency, or duration of the attack. An initial restriction of including participants with only moderate-to-severe pain was waived at the start of the title screening process because of fear of selection bias.

#### Intervention

The intervention was defined as bolus/infusion administration of ketamine of any dose or administration type (IV, IM, subcutaneous, intranasal, epidural, sublingual, rectal, oral). Studies in a perioperative setting and/or with mixed combinations of ketamine with another drug were excluded. There was no limit on the duration of treatment or number of treatments.

#### Comparator

Comparators included no treatment, placebo treatment, or conventional medical management, which could include pharmacological, physical, psychological, and/or interventional therapies.

#### Outcome

The primary outcome was the change in intensity of pain assessed on a numeric rating scale (NRS)/visual analog scale (VAS) at 60 min after the intervention. Secondary outcomes included (1) positive response (defined as a reduction in pain score by ≥30% from baseline at 60 min after the intervention) and effect of ketamine infusion on (2) pain intensity (NRS/VAS) at any time after the intervention up to 6 months posttreatment, (3) adverse effects, (4) functional outcome, (5) quality of life, (6) mood, and (7) patient satisfaction. The threshold of >30% pain relief has been demonstrated to constitute clinically meaningful improvement.^16^ The initial restriction of 6 months’ follow-up time was extended to 18 months at the time of data collection.

### Study Selection Process

All citations were independently screened on title and abstract for eligibility by two reviewers (Y.H. and P.K.) as per the inclusion criteria. Covidence^17^ was used as a systematic review management tool. Papers of interest were then full-text screened. Of the selected papers, data were independently extracted by two reviewers (T.M. and M.P.). Any disagreement was resolved through discussion with the senior author (P.K.).

### Data Extraction

The reference data, populations, and outcomes were extracted from the articles into prespecified tables on a standardized data collection form in Word that was pilot tested before use. Extracted data for each study included general characteristics (publication year, design, number of arms), patient characteristics (number, demographics, and sample size), clinical information (diagnosis, duration, pain intensity), details of intervention and comparator (dose and administration regimen), data on primary and secondary outcomes of interest, follow-up time points, and adverse effects.

### Assessment of Quality as Risk of Bias

Two review authors (Y.H. and M.P.) independently assessed the risk of bias for randomized controlled trials using the Cochrane Risk of Bias tool 2.0.^18^ Any disagreement was resolved through discussion with the senior author (P.K.). The Robins-I was used to assess the risk of bias in observational studies.^19^ For case reports/case series, the Quality Appraisal Tool for Case Series was used.^20^

### Data Synthesis and Analysis

We narratively synthesized the characteristics of all studies that met inclusion criteria. Study characteristics and treatment details were summarized. For continuous data, means (or medians) and standard deviations (or interquartile ranges or ranges) were extracted. No meta-analysis was performed because of the heterogeneity of data, low quality, and small sample sizes of included studies.

## Results

### Search Results

**Figure 1.**
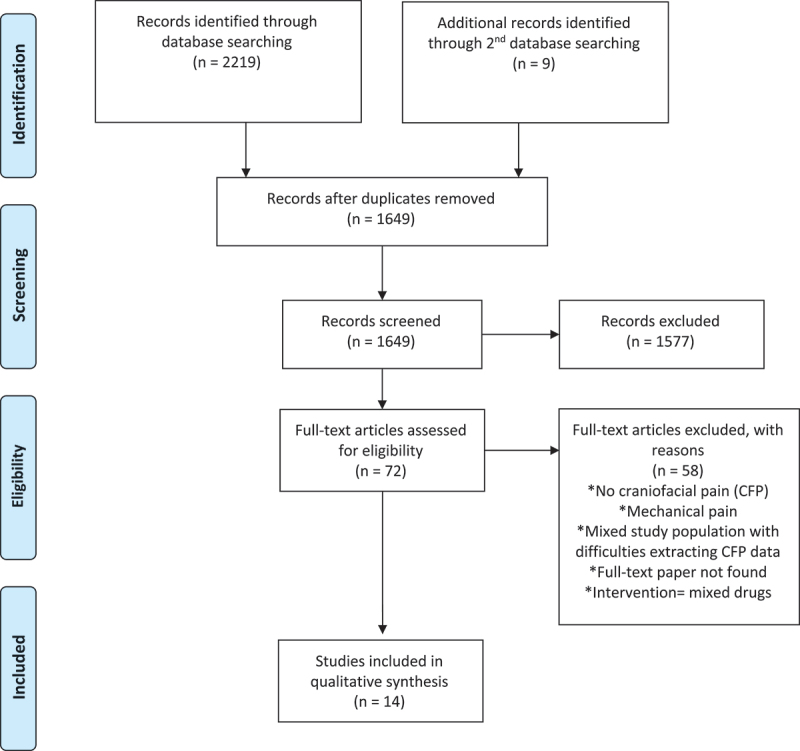
PRISMA flow diagram.

**Figure 2.**
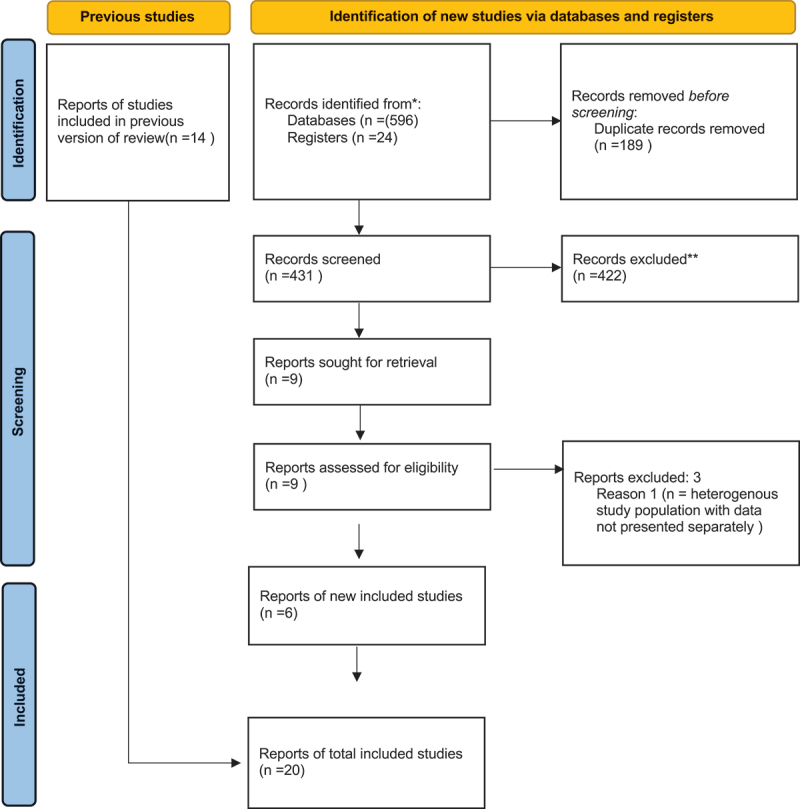
PRISMA flow diagram for updated systematic reviews.

A total of 2080 unique articles were retrieved from all searches, of which 1956 were excluded at the screening stage. During the initial search, 72 full texts were assessed for eligibility, of which 14 papers were deemed to meet all eligibility criteria for inclusion in this review (Figure 1). During the following searches, 9 additional full texts were selected and assessed, of which 6 papers met all eligibility criteria for inclusion in this review (Figure 2).

The 20 papers included in our review, reporting on 670 patients in total, included the following studies: Six randomized controlled trials (RCTs)^21–26^ and 14 observational studies, of which 5 were prospective cohort studies,^27–31^ 3 were retrospective cohort studies,^32–34^ 3 were case series,^35–37^ and 3 were case reports, were included^38–40^ (Tables 1 and 2).

**Table 1.** Characteristics of studies included in the systematic review: participants, interventions, comparators, and outcome measures.

Study (patient *n*)	Participants: Age, gender, diagnosis, duration of pain	Intervention, route, and dose	Comparator, route, and dose	Outcomes measured
**RCTs**				
Rabben et al.^21^ (*n* = 30) Crossover trial	Mean age (range): 57.6 years (29–89) F/M: 26/4 Diagnosis: Trigeminal neuropathy Mean duration of symptoms (range): 6 years (0.5–20) Baseline NRS ≥ 25/100	Week 1: One injection of IM K 0.4 mg/kg + midazolam 0.05 mg/kg Week 2: One injection of IM pethidine 1.0 mg/kg + midazolam 0.05 mg/kg Week 3: Oral K 4 mg/kg QHS × 3 days	Week 1: One injection of IM pethidine 1.0 mg/kg + midazolam 0.05 mg/kg Week 2: One injection of IM K 0.4 mg/kg + midazolam 0.05 mg/kg Week 3: Placebo × 3 days	Primary: NRS Secondary: Adverse effects
Afridi et al.^23^ (*n* = 18)	Mean age (range): 35 years K vs. 39 years C (18–55) F/M: 14/4 F: 77.8% K vs. 77.8% C Diagnosis: Migraine with aura Mean number of migraine-years: 14.6 years K vs. 21.9 years C Mean duration of aura (range): 30 h (18–72) K vs. 13 h (6–31) C Mean number of attacks per year: 21.7 K vs. 22.4 C Severity score without treatment: 9.5 K vs. 11 C	Intranasal K: One dose 25 mg (for three attacks) No treatment for three attacks	Intranasal midazolam: One dose 2 mg (for three attacks) No treatment for three attacks	Primary: Duration of aura attack Secondary: Severity of aura attack
Etchinson et al.^25^ (*n* = 34)	Mean age (SD): 38.5 years (13.75) K vs. 30.5 (8.3) C (range 18–65 years) F/M: 26/8 F: 81% K vs. 72% C Diagnosis: Migraine without or with aura Headache duration >24 h: 31% K vs. 33% C Migraine without aura: 47% K vs. 50% C Migraine with aura: 33% K vs. 17% C Probable migraine without aura: 13% K vs. 17% C Probable migraine with aura: 7% K vs. 17% C Baseline severe pain intensity: 88% K vs. 67% C	IV K 0.2 mg/kg; one dose	IV NS, equivalent amount in mL to IV K; one dose	Primary: Between-group difference in NRS score reduction from baseline to 30 min Secondary: Functional disability scores, categorical pain scores, pain, rescue medication, patient satisfaction, SERSDA side effects
Zitek et al.^24^ (*n* = 54)	Mean age (SD): 32.3 years (10.3) K vs. 37.4 years (10.4) C (range 18–65) Diagnosis: Primary headache at ER admission F/M: 38/16 F: 82.6% K vs. 78.3% C Mean (SD) baseline VAS: 77.8 (14.6) K vs. 78.3 (17.7) C Mean (SD) baseline VAS anxiety: 37.8 (35.7) K vs. 46.1 (36.4) C Mean (SD) baseline VAS nausea: 47.1 (33.0) K vs. 47.6 (35.2) C	K 0.3 mg/kg IV (diluted in 5 mL NS) + ondansetron 4 mg IV (diluted in 2 mL NS)	Prochlorperazine 10 mg IV (diluted in 5 mL NS) + diphenhydramine 25 mg IV (diluted in 2 mL NS)	Primary: Between-group mean VAS difference at 60 min Secondary: Changes in VAS pain at other FU times, admission rate, side effects, rescue medication use, patient satisfaction
Benish et al.^22^ (*n* = 53; 27/26)	Age (range): 31 years (25–42) K vs. 35 years (27–43) C (range 18–65 years) F/M: 37/16 F: 73.1% K vs. 66.7% C Diagnosis: Primary headache at ER admission Hx of migraine: 84.6% K vs. 81.5% C Mean (SD) baseline VAS: 73.5 (17.5) K vs. 74.5 (16.2) C Self-reported pain severity of >5/10	IV: 1000 mL NaCl + intranasal K: First dose 0.75 mg/kg Repeat dose: 0.25 mg/kg	IV: 1000 NS + 10 mg metoclopramide + 25 mg diphenhydramine + intranasal NS	Primary: Pain intensity at 30 mins Secondary: Rescue medication, side effects, hospital admission, return to care within 72 h, patient satisfaction
Sarvari et al.^26^ (*n* = 140)	Age: 41.6 ± 16.6 years F/M: 75/65 F: 55.3% K vs. 52.9% C Diagnosis: Primary headache (migraine, tension, and CH) Hx of headache: 67% K vs. 49% C Self-reported pain severity of ≥4/10 Mean (SD) baseline VAS: 8.0 (1.1) K vs. 9.0 (0.7) C (*P* < 0.05)	IV: 1000 mL NaCl + intranasal K: 0.75 mg/kg (maximum 75 mg)	IV: 30 mg ketorolac + intranasal NS (0.015 mL/kg, maximum 1.5 mL)	Primary: Pain intensity at 60 min Secondary: Side effects, vital signs
**Observational studies**				
Mathisen et al.^28^ (*n* = 7) P	Age range: 42–79 years F/M: 7/0 Diagnosis: Trigeminal neuropathy Duration: 1–21 years Baseline pain score: Severe	Bolus of IV/IM 0.4–1 mg/kg ± infusion of 0.25–0.5 mg/kg/30’	N/A	Primary: Pain intensity Secondary: Adverse effects
Rabben and Øye^27^ (*n* = 17) P	Age (range): 32–88 years F/M: 13/4 Diagnosis: Neuropathic orofacial pain Duration: 6 months–28 years Baseline pain score: NP	Bolus of IM K 0.4 mg/kg with 0.05 mg/kg midazolam, followed by 3 days of oral K 4 mg/kg QHS	N/A	Primary: Pain intensity Secondary: Adverse events
Granata et al.^29^ (*n* = 29) P	Mean age (SD): 44 years (NP) F/M: 2/27 Diagnosis: chronic or episodic CH Duration: 1–18 years Frequency of attacks: 2–8/day Episodic/chronic CH: 16/13 Baseline pain score: NP	IV infusion 0.5 mg/kg over 1 h, every 2 weeks. In total one to four treatments	N/A	Primary: Attack frequency Secondary: Attack intensity, responder rate
Pomeroy et al.^30^ (*n* = 77) R	Mean age (range): 40.6 years (16–65) F/M: 57/20 Diagnosis: CM: 63/77 NDPH: 14/77 MOH: 37/77 Median baseline pain score: 7.1	Mean length of ketamine infusion: 4.8 days (range 2–9) Mean K rate: 0.53 mg/kg/h (range 0.08–1.25)	N/A	Primary: Responder rate Secondary: Adverse events
Schwenk et al.^31^ (*n* = 61) R	Mean age (range): 42.4 years (20–65) F/M: 17/44 Diagnosis: Migraine: 59/61 (96.7%) CH: 2/61 (3.3%) Mean (SD) baseline pain score: 7.5 (0.2)	5-day IV infusion of 1 mg/kg/h max Mean length of infusion: 5.1 days (SD 0.1) Mean weight: 85.4 (SD 0.6) mg/h Mean infusion rate: 43.7 mg/h (SD 4.2)	N/A	Primary: Pain intensity; secondary: responder rate, adverse events
Petersen et al.^30^ (*n* = 23; 20/3)	Age: 49 (27–59) vs. 53 (30, 60) F/M: 5/15 vs. 2/1 Diagnosis: CCH Mean duration (years): 19 vs. 15 Median (range) baseline pain score: 10 (7–10) vs. 8 (7, 10)	Intranasal K 15 mg/dose; given every 6 min. Five doses max	Patient with CCH with no attack	Primary: >50% NRS reduction at 15 min after first dose Secondary: >50% NRS reduction at 30 min; effect on autonomic symptoms, rescue medication, adverse events
Schwenk et al.^33^ (*n* = 6) Pilot P	Age: 20–55 years F/M: 3/3 Diagnosis: Refractory chronic migraine Mean baseline pain score: 7.5 ± 2.2 C vs. 7.4 ± 1.4 K	5-day IV infusion K 1 mg/kg/h max Mean maximum K infusion rate on day 5: 72.5 ± 10.4 mg/h	5-day IV infusion IV lidocaine: 1 mg/min (max 4 mg/min) Mean lidocaine infusion rate on day 4: 2.5 ± 0.7 mg/min	Primary: Pain intensity Secondary: Rescue medications, side effects
Ray et al.^34^ *N* = 83 (33/50) R	Mean age: 42.9 ± 16.2 C vs. 42.2 ± 13.9 K F/M: 69/14 F/M: 78% C vs. 90.9% K Diagnosis: Migraine: 77/83 NDPH: 3/83 SUNCT: 2/83 CH: 1/83 Mean baseline VAS Migraine: 44.1 Mean number of previously failed preventative medications: 5.8 ± 3.8 C vs. 8.7 ± 4.1 K (*P* = 0.013) Mean number of comorbidities: 2.8 ± 2.5 C vs. 4.1 ± 2.9 (*P* = 0.024)	IV infusions: K started at 7 mg/h, max of 24 mg/h Mean duration: 5.1 (1.5) days	IV infusions: lidocaine: 2 mg/h mean duration: 6.2 (2.4) days	Primary: 50% or more reduction in pain intensity at the end of infusion Secondary: 30-day readmission, side effects
**Case series/case reports**				
Kaube et al.^35^ (*n* = 11)	Age range: 18–47 years F/M: 5/1 Diagnosis: FHM Baseline pain score: Severe	Intranasal K 25 mg; one dose at commencement of migraine aura 25 attacks were treated	N/A	Primary: Aura intensity Secondary: Time to cessation of aura, duration of aura, severity of motor deficit, presence or absence of visual hemifield disturbance and dysphagia, progression from one system to another
Lauritsen et al.^36^ (*n* = 6)	Median age (range): 36.5 (29–54) F/M: 5/1 Diagnosis: Chronic migraine without aura Median age at migraine onset (range): 17 years (8–29) Median duration of illness (range): 17 years (12–46) Mean number of failed acute migraine treatments (range): 18 (14–26) Mean baseline pain score: 9.5/10	IV K infusion of 0.1 mg/kg/h, increased by 0.1 mg/kg/h every 3–4 h as tolerated until the pain score of 3/10 was achieved, maintained for 8 h	N/A	Primary: Pain intensity Target end point: Achievement of VAS ≤3/10 for at least 8 h, timing of target point
Moisset et al.^38^ (*n* = 2)	45-year-old male Diagnosis: Chronic intractable CH Duration: 6 years, 6 attacks/day 28 years old male Diagnosis: CCH Duration: 6 years, 1–7 attacks/day	IV infusion 0.5 mg/kg over 2 h + MgSO_4_ IV infusion 3000 mg over 30 min	N/A	Primary: Attack frequency
Aggarwal^39^ (*n* = 1)	58-year-old male Diagnosis: SUNCT Duration: 6 years, 100 attacks/day	SC K infusion 6 mg/h × 6 days followed by SL 25 mg TID	N/A	Primary: Attack frequency Secondary: Medication use
Moisset et al.^37^ (*n* = 17)	Mean age (range): 35.2 ± 8.1 years (23–50) F/M: 3/14 Diagnosis: CCH Duration: 6.6 ± 4.3 years Mean number of daily attacks: 4.3 ± 2.4	IV K infusion 0.5 mg/kg × 2 h + MgSO_4_ 3 g	N/A	Primary: Number of daily attacks day 7 Secondary: Percentage of responders, adverse events, vital signs
Shiiba et al.^40^ (*n* = 1)	56-year-old male Diagnosis: SUNCT	IV K 0.4 mg/kg × 1 h × 7 days, then twice/month, then once/month	N/A	Primary: Attack frequency Secondary: Attack intensity, side effects

C = comparator; (C)CH = (chronic) cluster headache; CM = chronic migraine; ER = emergency room; FHM = familial hemiplegic migraine; FU = follow-up; Hx = history; K = ketamine; MgSO_4_ = magnesium sulfate; N/A = not applicable; NaCl = sodium chloride; NDPH = new daily persistent headache; NP = not provided; NS = normal saline; q = every; QHS = at bedtime; SC = subcutaneous; SERSDA = Side Effects Rating Scale for Dissociative Anesthetics; SL = sublingual; TID = three times daily; P = prospective; R = retrospective.

**Table 2.** Characteristics of studies included in the systematic review: Follow-up times, outcomes, and adverse effects.

Study (patient *n*)	Follow-up time points	Results: Primary outcome	Results: Secondary outcomes	Adverse effects
**Randomized controlled trials**				
Rabben^21^ (*n* = 30)	IM protocol: q1h for first day and at day 3 Oral protocol: 3 days	**K superior** At 60 min: pain relief with K > C: *n* = 9/26 VAS compared to baseline at 60 min: 63.2% ± 24.3 K vs. 76.9% ± 21.9 C (*P* < 0.02) Long-lasting (>12 h) pain relief with K > C: *n* = 8/26 Oral K: 5/8 long-lasting responders to IM K reported pain relief with oral K	N/A	K: Nausea, *n* = 2 (7.7%); dizziness, *n* = 18 (69.2%); sedation, *n* = 18 (69.2%); blurred vision, *n* = 16 (61.5%); xerostomia, *n* = 16 (61.5%); feelings of insobriety, *n* = 25 (96.1%); altered hearing, *n* = 4 (15.4%); hallucinations, *n* = 6 (23.1%) C: Nausea, *n* = 5 (19.2%); dizziness, *n* = 17 (65.4%); sedation, *n* = 14 (53.8%); blurred vision, *n* = 5 (19.2%); xerostomia, *n* = 17 (65.4%); feelings of insobriety, *n* = 12 (46.1%); altered hearing, *n* = 1 (3.8%); hallucinations, *n* = 0 Oral group: Adverse effects only in patients not asleep within 30 min of administration
Afridi^23^ (*n* = 18)	FU after six aura-attacks (three treated with intranasal K/midazolam, three treated with placebo)	**K superior** Duration of attacks: 3 h (2–46) shorter in K vs. placebo; 3 h (0–15) shorter in M vs. placebo	Severity of attack: K vs. placebo: difference was 1.5 (−0.5 to 2.5) on the clinical severity scale, suggesting baseline attack worse than treated attack M vs. placebo: difference was −0.5 (−0.7 to 1.5), suggesting that the attack was no different on treatment	K: 5 subjects described feelings of unreality, euphoria or mild giddiness Midazolam: 4 subjects reported transient sedation or giddiness
Etchinson^25^ (*n* = 34)	At 30 and 60 min	**No statistically significant difference between groups** Between-group median NRS difference at 30 min: 1.0 (IQR 0, 2.25; *P* = 0.0215) K vs. 2.0 (IQR 0, 3.75; *P* = 0.0034); ESD −1.0 (−2, 1.0), *P* = 0.5035	Mean categorical pain score change from baseline at 30 min: 0.56 (95% CI 0.44–0.68) K vs. 0.72 (95% CI 0.61–0.83) C; ESD 0.16 (95% CI −0.85–0.53) Mean functional disability score change from baseline at 30 min: 0.44 (95% CI 0.32–0.56) K vs. 0.39 (95% CI 0.30–0.48) C; ESD −0.05 (−0.59, 0.69) Patient satisfaction at 60 min: 62% K vs. 72% C Patient desires same treatment in the future: 62% K vs. 44% C Rescue medication requested at 30 min: 69% K vs. 78% C	SERSDA for generalized discomfort: Greater in K arm at baseline (*P* = 0.0247) and at 30 min (*P* = 0.008) SERSDA for fatigue: Greater in K arm at 60 min (*P* = 0.0216) No significant difference in side effect severity at 30 min between K and placebo arms 88% of K subjects had Ramsay score = 2 at 30 min
Zitek^24^ (*n* = 54)	At 15, 30, 45, and 60 min	**Comparator superior** Mean VAS score at 60 min: 34.3 K vs. 14.8 C; MD −19.5 (−37.8 to −1.3, *P* = 0.03)	Mean VAS score at 15 min: 38.7 K vs. 50.9 C; MD 12.2 (95% CI −6.2 to 30.7) Mean VAS score at 30 min: 45.8 K vs. 31.8 C; MD −14.1 (−31.1 to 3.0) Mean VAS score at 45 min: 39.8 K vs. 22.2 C; MD −17.6 (−35.7 to 0.59) Requirement for rescue medications: 47.8% K vs. 28.6% C; MD 19.2% (95% CI −7.1 to 45.6) Admission to hospital: 8.7% K vs. 3.6% C; MD 5.1% (95% CI −8.3 to 18.5) Satisfaction at FU (scale 0–10): 4.9 K vs. 8.3 C; MD 3.4 (95% CI 1.2–5.6) Headache at FU: 50% K vs. 30% C; MD 20% (95% CI −10.6 to 50.6)	Development of subjective restlessness: 13.0% K vs. 10.7% C; MD 2.3% (95% CI −15.6 to 20.2) Vomiting during collection period: 13.0% K vs. 7.1% C; MD 5.9% (95% CI −10.9 to 22.7) Patient withdrawal from study: K group: *n* = 2; prochlorperazine group: *n* = 1
Benish^22^ (*n* = 53;27/26)	In ED: at 15, 30, 60 min After ED: at 48–72 h post-ED discharge	**No statistically significant difference between groups** Mean VAS at 30 min: C 22.2 (SD 24) vs. K 29 (SD 21.6); ESD 6.8 (95% CI: −5.8 to 19.4)	Median VAS score at discharge: C 2 (range 0–4) vs. K 1 (range 0–4) ESD 0 (−1 to 1) Median VAS score at 72 h: C 0 (range 0–0) vs. K 0 (range 0–0); ESD (0–0) Required second dose: C 11.5% vs. K 51.9% Rescue medication: C 30.8% (14.3–51.8) vs. K 22.2% (8.6–42.3); ESD −8.5 (−33.2–16.1) Required admission for headache: C 11.5% (3.2–29.8) vs. K 3.7 (0.0–19.8); ESD −7.3 (−22.5 to 6.9) Returned to ED within 72 h: C 11.5 (3.2–29.8) vs. K 0 (0–14.8); ESD −11.5 (−24.1 to 1.0 In control arm, 80.8% received ketorolac and 26.9% received DXM Patient satisfaction: C 9 (8–10) vs. K 9 (7–10); ESD (−1 to 1)	Adverse event rate: 66.7% (CI 46–83.5%) K vs. 65.4% (95% CI 44.3–82.8) C
Sarvari^26^ (*n* = 140)	At 30, 60, and 120 min	**K superior** At 60 min: Mean VAS K < C: K 2.07 ± 0.2 vs. C 2.42 ± 0.7 (*P* < 0.001)	At 30 min: Mean VAS K < C: K 3.47 ± 0.5 vs. C 4.98 ± 0.7 (*P* < 0.001) At 120 min: Mean VAS K = C: K 1.10 ± 0.3 vs. 1.00 ± 0.0	Dizziness: K 11 (15.71%) vs. C 2 (2.86%), *P* = 0.009 Nausea: K 8 (11.43%) vs. C 2 (2.86%), *P* = 0.049 Heart rate rising: K 28 (40%) vs. C 11 (15.71%), *P* = 0.001 Blood pressure rising: K 9 (12.86%) vs. C 23 (32.86%), *P* = 0.005
**Observational studies**				
Mathisen,^28^ (*n* = 7) P	q15min until 80 min posttreatment	**No change** Two patients: Pain-free for 1 day posttreatment One patient: Pain-free for 3 days posttreatment Four patients: No treatment effect	N/A	Blurred vision, 6 (85.7%); altered hearing, 4 (57.1%); dizziness, 7 (100%); illusions, 4 (57.1%); dreams, 3 (42.9%); hallucinations, 3 (42.9%) All side effects subsided 30 min after treatment
Rabben,^27^ (*n* = 17) P	30 min after IM dose 1 day after oral dose	**Improved** IM K: 4/17 patients: no analgesia; 7/17: <60 min: analgesia (not quantified); 6/17: > 60 min: mean pain reduction 80% Oral K: 5/13: no analgesia Responders to IM also responded to oral dose	N/A	IM K: 17/17 had some level of side effects: sedation, blurry vision, dry mouth. 4/17 dropped out due to side effects Oral K: 4/13
Granata,^29^ (*n* = 29) P	At 18 months	**Improved** CCH: 7/13: complete resolution of attacks for 3–18 months. Onset: 1–2 weeks after final treatment; 4/13: no improvement; 1/13: excluded due to spontaneous symptom improvement before intervention Episodic CH: 16/16: complete cessation of attack	CCH: 1/13: partial responder: HA decreased in intensity K significantly shortened subsequent episodes for all patients	1/29: Dissociation 1/29: Hypotension 2/29: Bradycardia 29/29: Moderate-to-severe fatigue after infusion lasting up to 24 h
Pomeroy,^32^ (*n* = 77) R	At end of infusion At 1 month	**Improved** NDPH: Acute responders: 8/14, MD 4.25 (95% CI 2.59–5.91, *P* < 0.0005) Sustained responders: 4/6, MD 2.5 (95% CI 0.91–4.09, *P* < 0.0154) CM: Acute responders: 47/63, MD 4.628 (95% CI 3.99–5.27, *P* < 0.0001) Sustained responders: 11/36, MD 2.136 (95% CI 0.91–3.37, *P* < 0.0031)	N/A	Adverse events common: 66/77 28/77 diplopia, 19/77 confusion, 16/77 hallucinations, 9/77 dysarthria, 9/77 dizziness, 7/77 vivid dreams, 7/77 sedation, 6/77 unsteady gait, 5/77 worsened nausea, 1/77 nonepileptic seizure, 1/77 transaminitis, 1/77 suicidiality, 2/77 fall Most events stopped after stop infusion
Schwenk,^31^ (*n* = 61) R	At discharge At 30 and 90 days	**Improved** Mean NRS: 7.5 (SD 0.2) vs. 3.4 (SD 0.3) (baseline vs. discharge), *P* < 0.001	Baseline NRS: 7.8 (SD 0.23) immediate responder vs. 6.8 (SD 0.64) nonresponder Posttreatment NRS: 2.6 (SD 0.28) immediate responder vs. 6.6 (SD 0.68) nonresponder, *P* < 0.01 Mean time to lowest NRS: 4.56 h First FU at 38.1 days (SD 4.7): 21/52 (40%): sustained response; 30/51 (58%): no longer response; 1/51: no immediate response but pain relief at 1 month Second FU 101.3 days (SD 8.8): 19/49 (39%) sustained response No significant difference in age/sex between responders vs. nonresponders	Immediate responders: 53/61 nystagmus; 31/61 sedation; 23/61 N/V; 23/61 blurred vision; 17/61 hallucinations; 8/61 vivid dreams No significant difference in adverse effects
Petersen,^30^ (*n* = 23; 20/3) Proof of Concept	At 1, 3, 6, 30, 60, 120, 180 min At 1–2 weeks	**Improved** At 15 min: Mean difference 1.1 (95% CI: −0.6 to 2.7, *P* = 0.188); 4/20 patients >50% pain relief	At 30 min: Mean difference: 4.3 (95% CI: 2.4–6.2, *P* > 0.001) Complete relief for 8/20 patients (timing not known) 6/20: no relief 50% of patients preferred K over oxygen/sumatriptan	Adverse event rate: 85% Most common: dizziness, light-headedness, nausea/vomiting, paresthesias
Schwenk,^33^ (*n* = 6) Pilot P	At end of treatment At 1 month	**Improved** Lidocaine: Mean NRS by end of hospitalization/treatment (day 5) for lidocaine: 4.7 ± 2.8 (*P* ≤ 0.05) Mean NRS at postdischarge visit at 28 ± 8 days after treatment for lidocaine: 7.0 ± 1.4 (*P* > 0.05) K: Mean NRS at baseline for K: 7.4 ± 1.4 Mean NRS by end of hospitalization/treatment (day 5) for K: 3.7 ± 2.3 (p ≤ .05) Mean NRS at postdischarge visit at 4.1 ± 7 days after treatment for K: 7.2 ± 1.7 (*P* > 0.05) Mean NRS decrease K > C: K −3.7 vs. C −2.8 (*P* ≤ 0.05)		No serious adverse events for K or C Lidocaine: 4/6 experienced transient adverse events (1 with bradycardia/junctional heart rhythm and nausea, 1 hallucinations and blurry vision, 1 nausea and blurry vision, 1 insomnia) K: 6/6 experienced adverse events including hallucinations, nightmares, vivid dreams, blurry vision, and nausea/vomiting
Ray,^34^ *N* = 83 (33/50) R	At end of infusion At 4 weeks	**Improved** Migraine cohort (*n* = 77): *n* = 45 C vs. *n* = 32 K Responder: 23/45 (51.1%) after 4.5 ± 0.5 days C vs. 11/32 (34.4%) after 3.4 ± 0.5 days K Pain-free: 14/45 (31.1%) after 6.2 ± 2.4 days C vs. 5/32 (15.6%) after 5.1 ± 1.5 days K Mean VAS percentage (SD) reduction: 53% (39.3) C vs. 31.6% (41.3) K NDPH cohort (*n* = 3): *n* = 2 C vs. *n* = 1 K Responder: 2/2 C vs. 0/1 K Pain-free: 0/2 C vs. 0/1 K	Migraine cohort (*n* = 77): 30-day readmission: 3/45 (6.7%) C vs. 1/32 (3.1%) K NDPH cohort (*n* = 3): 30-day readmission: 0/2 C vs. 0/1 K	K: Anxiety/agitation/dizziness,3%; confusion, 6.1%; paresthesias, 12.1%; hallucinations, 6.1%; psychogenic seizure, 3% C: Anxiety/agitation, 8%; dizziness, 6%; arrhythmia, 4%; hypotension, 6%; confusion, 2%; paresthesias, 2%; rash, 2%; nausea, 2%
**Case series/case reports**				
Kaube,^35^ (*n* = 11)	q15min following onset of aura until cessation	**Improved** 5/11 patients: Improvement in aura symptoms using K for all attacks 6/11 patients: No benefit	2/11 patients: Improvement in headache severity	6/11 patients: Feelings of alienation and mild ataxia
Lauritsen,^36^ (*n* = 6)	At end of infusion	**Improved** All patients achieved the target end point (VAS ≤3/10 for at least 8 h)	Mean time (range) to target end point: 44 h (12–82) Mean (range) K infusion rate at pain relief end point: 0.34 mg/kg/h (0.12–0.42)	One patient experienced a brief dissociative experience; resolved with reduction in infusion rate
Moisset,^38^ (*n* = 2)	At 6 weeks	**Improved** Patient 1: Complete resolution of attacks until 6 weeks Patient 2: 50% decrease in attack frequency for 6 weeks	N/A	None
Aggarwal,^39^ (*n* = 1)	At 3 months	**Improved** Attack-free for 3 months At 3 months: Slight recurrence in attacks, managed with temporary increase in SL K	D/C other medications (pregabalin, tapentadol, lamotrigine, amitriptyline)	No reported side effects
Moisset,^37^ (*n* = 17)	At 7 days	**Improved** Mean number of daily attacks decreased from 4.3 ± 2.4 to 1.3 ± 1.0, MD −2.75 (95% CI −4.0 to −1.75, *P* < 0.001)	Time to analgesic effect: 1 to 6 days (median = 3 days) Length of analgesic effect: 2 to 68 weeks (median = 4 weeks) Responders: 13/17 (76.5%, 95% CI 56.3–96.6) Full resolution: 7/17	Transient sedation: 7/17 (41.2%) No patients experienced hallucinations No debilitating sedation 1 h after end of infusion No bradycardia or high blood pressure
Shiiba,^40^ (*n* = 1)	At end of infusion At 3 months	**Improved** No SUNCT pain attacks for 3 months after treatment	No side effects	None

C = comparator; (C)CH = (chronic) cluster headache; CI = confidence interval; CM = chronic migraine; D/C = discontinued; DXM = dexamethasone; ED = emergency department; ESD = effect size difference; FU = follow-up; HA = headache; IQR = interquartile range; K = ketamine; M = midazolam; MD = mean difference; N/V = nausea and vomiting; SERSDA = Side Effects Rating Scale for Dissociative Anesthetics; SL = sublingual; P = prospective; R = retrospective; Comparator = prochlorperazine + diphenhydramine.

### Risk of Bias for Included Studies

The results for assessment of the risk of bias for the included RCTs are listed in Appendix 3. The overall risk of bias was deemed to have “some concerns” for two of the RCTs^24,25^ and “high risk of bias” for the remaining four RCTs.^21–23,26^ The risk of bias assessment of the included nonrandomized trials showed one study of low risk of bias,^30^ one study of moderate risk of bias,^33^ two studies deemed to have a serious risk for bias,^27,32,34^ and two studies that demonstrated a critical risk of bias^28,29,31^ (Appendix 4). The quality of the two case series was moderate^35,36^ and one was of high quality^37^ (Appendix 5).

### Study Characteristics

Demographic and clinical characteristics of the 670 patients reported in the 20 included studies are summarized in Table 1. The use of ketamine for the treatment of primary headache was discussed in 17 studies (*n* = 616), including 5 RCTs^22–26^ and 12 observational studies (6 nonrandomized trials^29–34^ and 6 case series/case reports).^35–40^ Three studies (*n* = 54) reported the use of ketamine for treatment of neuropathic facial pain, including 1 RCT^21^ and 2 prospective observational studies.^27,28^

#### Primary Headaches

##### Demographics and Pain Profiles of Participants in the Included Studies

We found five RCTs including 299 patients with primary headaches: migraine, tension-type headaches, and cluster headaches (63% females, 18–65 years) (Table 1).^22–26^ Baseline pain intensity ranged from VAS of 56/100 to 95/100.^22,24–26^

We found 12 observational studies including 317 patients (50% females) with the following diagnoses: chronic migraine (CM; *n* = 222), cluster headache (CH)–episodic (*n* = 17) or chronic (CCH; *n* = 57), short-lasting unilateral neuralgiform headache with conjunctival injection and tearing (SUNCT; *n* = 4), medication overuse headache (MOH; *n* = 37), and new daily persistent headache (NDPH; *n* = 17).^29–40^ Baseline pain intensity was moderate to severe in all observational studies. The duration of headache history was only provided in 3 studies.^23,30,37^

##### Details of the Ketamine Treatment

###### Route of Administration and Dosing

Treatment with a single^23–26,31^ or double^22^ or five^30^ boluses of ketamine was investigated in seven papers. Five papers (three RCTs, one cohort study, one case series; *n* = 245) looked into the effect of and *intranasal* bolus of ketamine compared to midazolam,^23^ diphenhydramine,^22^ ketorolac,^26^ or no comparator.^30,35^ Bolus dose ranged from 0.25 to 0.75 mg/kg. Ketamine was administered as an IV bolus in two RCTs.^24,25^ Bolus dose ranged from 0.2 to 0.3 mg/kg and comparators were placebo^25^ or prochlorperazine with diphenhydramine.^24^

The effect of IV ketamine infusions was reported in ten observational studies (including 283 patients).^29,31–34,37,40^ Patient population in these studies was mixed and included patients with CM and CH. Infusions were given over dosages ranging from 0.1 to 1 mg/kg/h over various durations (ranging from a single 1-h infusion up to infusions for up to 6 h on nine consecutive days). Two studies had a comparator (lidocaine).^31,34^

###### Duration of Follow-Up

The follow-up time in the RCTs ranged from 60 min^25,26^ to 72 h posttreatment,^22,24^ with one additional trial specifying only that follow-up occurred for six consecutive migraine attacks.^23^ The follow-up time for the observational studies was longer, ranging from immediately posttreatment,^30,34–36^ to 1 week,^37^ 1 month,^31,32^ 3 months,^33,38–40^ and 18 months^29^ after treatment.

##### Outcome

###### Impact on Migraine Pain Intensity

Four studies (all RCTs) investigated the analgesic effect of ketamine bolus treatment on migraine intensity,^22,24–26^ and five studies (all observational studies) investigated ketamine infusions.^31–36^

Of the four RCTs investigating the analgesic efficacy of ketamine bolus treatment, only one RCT demonstrated superiority of ketamine compared to the comparator (ketorolac), providing significant pain relief up to 2 h posttreatment.^26^ The three other RCTs failed to demonstrate superiority of ketamine compared to placebo, metoclopramide, or midazolam^22,23,25^ (Table 2).

In stark contrast, all of the five observational ketamine infusion studies (*n* = 233) reported significant pain improvement at the end of the infusion period.^31–34,36^ Infusions of solely ketamine were given for 8 h^36^ to 5 days in a row.^32,33^ In two studies, the effect of multiday ketamine infusion was compared to multiday lidocaine infusion.^31,34^ In the studies with lidocaine as a comparator, both groups were associated with significant pain reduction at the end of the infusion as compared to baseline, although the difference between groups was significant in favor of lidocaine in one study^34^ and in favor of ketamine in the other study.^31^

Only three out of five papers reported long-term outcomes. Two studies noted a positive effect up to 1^32^ and 3 months.^33^ In the third paper, patients had returned back to their baseline pain at 1-month follow-up.^31^

###### Impact on Aura Attack during Migraine

Only one RCT investigated the effect of ketamine and midazolam on length and severity of aura compared to placebo.^23^ The severity of the aura was significantly improved by ketamine compared to placebo. However, both agents were equally effective in reducing the median duration of the attack compared to placebo. In a case series of 11 patients with familial hemiplegic migraine treated with 25 mg intranasal ketamine, 5 patients experienced an improvement of aura duration and severity for all 14 attacks treated. Three patients experienced a return of aura symptoms after initial improvement.^35^

###### Impact on Pain Relief and Attack Frequency of TAC Headaches

All seven studies including patients with TAC (*n* = 76) reported a resolution or reduction in the attack frequency after ketamine treatment. An immediate but short-lasting effect was noted after bolus treatment^30^ and an immediate to sustained effect was noted after ketamine infusion.^29,34,37–40^ Patients were reported to have more than a 50% reduction in attack frequency for 6 weeks^38^ to complete resolution of attacks for 6 weeks^34,37,38^ up to 3 months.^39,40^

###### Effect on Pain-Associated Domains (Functional Outcome, Sleep, Quality of Life)

Only one RCT reported on the effect of ketamine on pain-associated domains. No significant difference in functional disability scores (rated on a 4-point scale), measured 30 min posttreatment, between the IV ketamine bolus and placebo groups was identified.^25^

###### Patient Preference/Satisfaction

Only three papers reported on patient satisfaction and/or preference for ketamine versus the comparator using an NRS.^22,24,25^ In only one study was a significant difference identified, in favor of prochlorperazine.^24^

###### Impact of Ketamine on Intake of Pain Medication

A few studies commented on change in use of rescue medication during or following ketamine treatment. Although not statistically significant, two RCTs demonstrated that a smaller proportion of patients in the ketamine group needed rescue medication during the infusion compared to the comparator.^22,25^ Two case series mentioned a reduction in dose and/or discontinuation of analgesics following ketamine infusion, but further details were not provided.^38,39^

###### Adverse Effects and Complications of Ketamine Infusion

Approximately 70% of patients across all studies experienced side effects related to ketamine infusions; however, most were mild and resolved with decreased rate or ending the ketamine infusion. Side effects included dizziness, sedation, and blurred vision’ One patient’s ketamine infusion was stopped because the patient experienced suicidal thoughts^32^ and one patient preferred to stop ketamine because of the side effects.^33^ One additional patient developed an asymptomatic elevation in liver enzymes.^32^ None of the patients needed to be hospitalized because of intolerable side effects such as feelings of insobriety, confusion, nausea and vomiting, hallucinations, and tachycardia.

#### Facial Pain

##### Demographics and Pain Profiles of Participants in the Included Studies

Of three studies including 54 patients (85% females, 29–89 years), one crossover RCT (*n* = 30)^21^ and one small observational study (*n* = 7)^28^ examined the use of ketamine for patients with trigeminal neuropathy. Another small observational study (*n* = 17) included patients with neuropathic orofacial pain (not specified).^27^ Baseline pain intensity was moderate to severe^21,28^ or not provided.^27^ The duration of pain ranged from 6 months to 28 years.

##### Details and Outcome of Ketamine Treatment

In the three studies, all patients received a single bolus of ketamine 0.4 mg/kg. One study compared ketamine to IM pethidine 1 mg/kg.^21^ In two studies, the bolus treatment was followed by 4 days of oral ketamine.^21,27^ Follow-up duration was short in these studies and ranged from 60 min after ketamine IM bolus^21,27,28^ to 3 days after oral treatment.^21,27^ Ketamine demonstrated a significant immediate improvement of facial pain in two studies for the duration of follow-up (3 days).^21,27^ It was noted that those responding to IM ketamine also reported pain relief with oral ketamine.^21,27^ The adverse events profile was similar as reported in the primary headache studies.

## Discussion

Despite recent evolutions in pharmacological and interventional management of patients with CFP, many patients continue to experience a significant reduction in quality of life, thereby imposing a significant burden on the health care system. We therefore conducted this systematic review to evaluate the evidence on the role of ketamine for patients with headache and/or facial pain.

### Impact on Headache and Facial Pain

Although we evaluated 5 RCTs and 12 observational studies, evidence remains conflicting on the efficacy of ketamine for treatment of primary headaches. This is likely due to the limited study quality, moderate to high risk of study bias, confounding and substantial heterogeneity across studies in terms of study design, patient populations, details of ketamine treatment, and (lack of) follow-up. Only 2 of the 5 RCTs demonstrated a significant immediate effect of ketamine on the intensity of pain and aura of migraine relief in the emergency room,^23,26^ and the remaining RCTs failed to demonstrate a significant effect of ketamine on headache intensity compared to various comparators. On the other hand, all observational studies demonstrated a significant reduction in migraine pain intensity and duration, as well as decreased frequency and intensity of CH attacks immediately postintervention that lasted for up to 3 months postinfusion. The difference in these findings may be explained as follows. First, it may be attributed to the shorter administration times and/or lower dose of ketamine bolus treatment in the RCTs, because nearly all observational studies explored single-/multiple-day ketamine infusions. The typical doses for chronic pain IV ketamine treatment are 0.2 to 0.75 mg/kg (bolus), 0.5 to 2 mg/kg/h (infusion), 0.1 to 0.5 mg/kg for IM administration, and 0.2 to 1 mg/kg for intranasal administration, which is higher than what was administrated in the RCTs.^10^ A high dosage and extended administration (infusions) of ketamine have been associated with better pain relief in patients with chronic pain.^41^ Similar conclusions were made in the consensus guidelines on the use of IV ketamine infusions for chronic pain.^10^ Future research in patients with primary headache should therefore focus on investigating the effect of high-dose and repeated administration of ketamine in a randomized controlled setting. Second, most RCTs had a mixed patient population, including patients with acute and chronic headache of migraine, cluster, and MOH types, which increased study heterogeneity, potentially affecting the outcome. Lastly, the 5 papers that did show a positive outcome of ketamine bolus treatment investigated patients solely with TAC, and bolus treatment failed in studies on patients with chronic migraine. This difference in outcome could be explained by a difference in pathophysiology, where TAC is driven by changes in the sphenopalatine ganglion, which potentially is more susceptible for the ketamine effect.

The evidence on ketamine for neuropathic facial pain is scarce but appears promising. Intramuscular ketamine was associated with significant improvement in two out of three studies on patients with trigeminal neuropathy,^21^ and a tendency for continued pain relief with oral ketamine after initial IV/IM treatment was also noted.^21,27^ This treatment option of oral ketamine should be further explored because this could be a valuable therapy for patients in remote areas with limited access to health care facilities.

### Effect on Pain-Associated Domains

Although the *International Classification of Headache Disorders* third edition diagnostic criteria for CFP are widely accepted and recommended,^42^ a majority of the papers did not mention use of this validated instrument for diagnosis. This is a weakness that needs to be addressed to avoid misclassification bias and improve generalizability of the study results. Though a handful of studies examined patient satisfaction/preference and the need for rescue medications, most papers in this review did not mention use of validated tools to evaluate the effect of ketamine on pain-associated domains (sleep, mood, quality of life) in addition to its analgesic efficacy. This is surprising because the importance of evaluating pain-related domains in clinical and research settings is established, and validated tools to evaluate these domains are available.^43^ Ketamine has been demonstrated to have an effect on sleep and mood and could therefore potentially improve patients’ quality of life even if pain intensity is unchanged.^44,45^

### Safety

It is widely accepted that ketamine is associated with adverse psychomimetic, cardiovascular, and gastrointestinal effects resulting from its activity on a variety of substrate receptors including NMDA, acetylcholine, opioid, monoamine, and histamine.^10^ The incidence of reported ketamine-induced side effects in the included papers was high regardless of type of administration or dose and only included psychomimetic effects. Most side effects were mild and resolved after decreasing the dose or ending treatment. Although central nervous system effects appear to be dose dependent when ketamine is used in anesthetic doses, the evidence is not as clear for subanesthetic regimens, beyond a yet-to-be-determined threshold.^10^ In our review, no clear difference was noted in the incidence of side effects when comparing high to low doses. Most patients experienced hallucinations, which resolved after cessation of treatment, and one patient experienced suicidal thoughts. Based on the America Psychiatric Association^44^ guidelines on the use of ketamine, a history of psychosis is a contraindication for administration of subanesthetic IV ketamine.

### Strengths and Limitations

We found one other systematic review on the efficacy of ketamine for headaches, with evidence found in RCTs that were primarily focused on bolus administration of ketamine for acute headache pain relief.^46^ The authors concluded that the benefit of ketamine for headache treatment is unclear but that long-term follow-up and different ketamine dosages in patients with chronic pain should be explored. Therefore, our systematic review attempted to take a closer look at the larger amount of evidence in observational trials that focused on ketamine infusions. Our review demonstrated that prolonged ketamine infusions can indeed significantly and sustainably reduce migraine intensity and frequency of CH attacks. This provides promising evidence, and this temporal and dose-dependent relationship should be further explored in new high-quality trials. To our knowledge, no attempt has been made to systematically gather evidence regarding the effects of ketamine on facial pain.

This review has several limitations. Most of the studies included in this review reported observational data, with inherent high risk of confounding and bias. Further, the heterogeneity across papers was substantial, with large differences in dosing, duration and route of administration, and comparators, which made comparison challenging and precluded a meta-analysis. Lastly, this review did not assess the long-term benefits and harms of ketamine for the treatment of CFP owing to lack of data in the literature.

A high-quality placebo-controlled RCT investigating the effect and safety of high-dose and/or prolonged ketamine infusion treatment on refractory headache pain and pain-associated domains would be the logical next step to investigate ketamine’s potential in the treatment of CFP. Recent systematic reviews and meta-analyses of this evidence show that infusions of ketamine 1 mg/kg/h are associated with greater and sustained pain relief compared to one-off bolus and low-dose infusion regimens.^10^ However, adverse effects of ketamine are common and could limit dose escalation, and the potential long-term risks of repeated administration of high doses of ketamine need to be further investigated.

## Conclusions

Current evidence on the efficacy of ketamine for treatment of CFP remains conflicting, precluding the ability to make any recommendations. This is likely due to the limited study quality, moderate to high risk of study bias, and substantial heterogeneity across studies in terms of study design, patient populations, details of ketamine treatment, and (lack of) follow-up. Ketamine bolus treatment showed significant reduction of migraine aura severity, TAC intensity and frequency, and trigeminal neuropathic pain but failed to reduce migraine intensity or duration. The included observational trials suggest that ketamine infusion treatment has an immediate and sustained benefit on headache pain intensity, possibly because of the prolonged duration of treatment and higher dose of administration. Most papers failed to evaluate the effect of ketamine on pain-associated domains. RCTs are required that focus on the dose–response relationship and immediate and long-term effects of prolonged ketamine treatment on CFP and pain-associated domains.

## Supplementary Material

Supplemental Material

Supplemental Material

Supplemental Material

Supplemental Material

Supplemental Material

Supplemental Material
