# Supplementary material for: A systematic review of the efficacy of ketamine for craniofacial pain
Source: Can J Pain. 2023 Jun 26;7(1):2210167. doi: 10.1080/24740527.2023.2210167 (PMC10294769; doi:10.1080/24740527.2023.2210167)
Supplement: Supplemental Material [file UCJP_A_2210167_SM0818.docx]

| Author, year | 1 | 2 | 3 | 4 | 5 | 6 | 7 | 8 | 9 | 10 | 11 | 12 | 13 | 14 | 15 | 16 | 17 | 18 | 19 | 20 |
| --- | --- | --- | --- | --- | --- | --- | --- | --- | --- | --- | --- | --- | --- | --- | --- | --- | --- | --- | --- | --- |
| Kaube^34^, 2000 | - | + | - | +/- | + | + | +/- | - | - | + | - | + | + | - | +/- | - | - | + | - | + |
| Lauritsen^35^, 2016 | + | - | - | + | + | + | + | + | + | + | - | + | + | - | + | - | - | + | + | + |
| Moisset^36^, 2020 | + | + | + | + | + | + | - | + | + | + | +/- | + | + | + | +/- | + | + | + | + | + |

+ = yes - = no +/- = unclear

**Table 5.** Quality Appraisal Checklist for Case Series Studies (19)
