# Supplementary material for: A systematic review of the efficacy of ketamine for craniofacial pain
Source: Can J Pain. 2023 Jun 26;7(1):2210167. doi: 10.1080/24740527.2023.2210167 (PMC10294769; doi:10.1080/24740527.2023.2210167)
Supplement: Supplemental Material [file UCJP_A_2210167_SM0817.docx]

| **Author, year** | **Confounding** | | **Participant Selection** | | **Classification of Interventions** | | **Deviations from Intended Interventions** | | **Missing Data** | | **Outcome Measures** | | **Outcome Reporting** | | **Overall Risk of Bias** | |
| --- | --- | --- | --- | --- | --- | --- | --- | --- | --- | --- | --- | --- | --- | --- | --- | --- |
|  | RoB | Direction | RoB | Direction | RoB | Direction | RoB | Direction | RoB | Direction | RoB | Direction | RoB | Direction | RoB | Direction |
| **Mathisen^27^1995** | -- | AN | -- |  | -- | AN | - |  | --- | TN | -- | AN | -- | TN | --- |  |
| **Rabben^26^, 2001** | -- | AN | + |  | + |  | -- | AN | - | AN | - | AN | + |  | -- | AN |
| **Granata^28^, 2016** | --- | AN | - |  | + |  | -- | AN | --- | AN | -- | AN | -- | AN | --- | AN |
| **Pomeroy^31^, 2017** | -- | AN | + |  | + |  | + |  | + |  | -- | AN | + |  | -- | AN |
| **Schwenk^30^, 2018** | + |  | + |  | + |  | - | AN | - |  | - | AN | + |  | - | AN |
| **Petersen^29^,**  **2021** | + |  | + |  | + |  | ++ |  | + |  | + |  | + |  | + |  |
| **Schwenk^32^, 2021** | -- |  | - |  | + |  | + |  | + |  | --- |  | --- |  | --- |  |
| **Ray^33^,**  **2022** | -- |  | - |  | - |  | - |  | - |  | - |  | - |  | -- |  |

+ = low risk - = moderate risk -- = serious risk --- = critical risk

TN = towards null AN = away from null

**Table 4.** The Risk of Bias in Non-Randomized Studies of Interventions (ROBINS-I) assessment tool (18)
