# Supplementary material for: A systematic review of the efficacy of ketamine for craniofacial pain
Source: Can J Pain. 2023 Jun 26;7(1):2210167. doi: 10.1080/24740527.2023.2210167 (PMC10294769; doi:10.1080/24740527.2023.2210167)
Supplement: Supplemental Material [file UCJP_A_2210167_SM0815.docx]

| Author, year | Domain 1 | Domain 2 | | Domain 3 | Domain 4 | Domain 5 | Overall Risk of Bias |
| --- | --- | --- | --- | --- | --- | --- | --- |
|  | **Randomization Process** | **Assignment to Intervention** | **Adherence to Intervention** | **Missing Outcome Data** | **Measurement of Outcome** | **Outcome Reporting** |  |
| Rabben^20^, 1999 | - | + | + | +/- | + | - | - |
| Afridi^22^, 2013 | + | + | +/- | - | - | + | - |
| Etchinson^24^, 2017 | + | + | + | + | + | +/- | +/- |
| Zitek^23^, 2017 | + | + | +/- | + | + | + | +/- |
| Benish^21^, 2019 | + | - | +/- | + | + | + | - |
| Sarvari^25^, 2022 | + | +/- | +/- | - | + | + | - |

+ = low risk - = high risk +/- = some concerns

**Table 3.** Cochrane Risk of Bias Tool for RCTs 2.0 (17)
