# Supplementary material for: A systematic review of the efficacy of ketamine for craniofacial pain
Source: Can J Pain. 2023 Jun 26;7(1):2210167. doi: 10.1080/24740527.2023.2210167 (PMC10294769; doi:10.1080/24740527.2023.2210167)
Supplement: Supplemental Material [file UCJP_A_2210167_SM0798.docx]

**
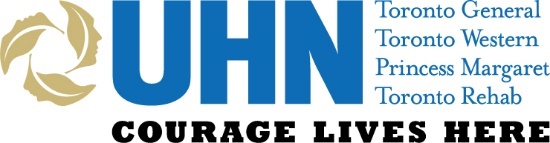
UHN Health Sciences Libraries**

# Literature Search Results

**#1439; KS Sys Rev : UPDATE September 2022: Headache or Facial Pain or Craniofacial Pain and Ketamine**

For: Drs Yasmine Hoydonckx & A Bhatia

Department: Anesthesia

Date Completed: Monday, Sept. 26, 2022

Tel:

Fax:

Email:

**Attached is your search for**:

1. **Update Systematic Review search** for: Headache OR Facial Pain OR Craniofacial Pain and Ketamine; limited to human. **Updated from November 1, 2020 to present, where possible**

**The databases searched were**:

1. [MEDLINE](#Medline); 2. [Medline In-Process/ePubs](#Medline_In_Process); 3. [Embase](#Embase); 4. [CCTR](#CCTR); 5. [CDSR](#CDSR); 6. [Web of Science](#Web_of_Science); 7. [Scopus](#Scopus); 8. [ClinicalTrials.Gov](#ClinicalTrials_Gov); 9. [WHO ICTRP](#WHO_ICTRP).

**RESULTS & STRATEGY USED**: *see following*

**Search Completed By:** Marina Englesakis, Information Specialist

You may contact me either by telephone at (416) 340-4800 x3022 or via e-mail at [*marina.englesakis@uhn.ca*](mailto:marina.englesakis@uhn.ca)*.*

It is important that you are satisfied with your search results.

If you have any questions regarding this search, or if the results were not satisfactory, please do not hesitate in contacting me.

To request items not available in our library system, an INTERLIBRARY LOAN REQUEST FORM can be obtained from the library’s circulation desk or through the Virtual Library (<http://www.uhn.ca/Education/libraries/ill.asp> ). Any questions regarding our Document Delivery Service can be directed to Caleb by telephone at 416-340-4121 or by email at [Caleb.Nault@uhn.ca](mailto:Caleb.Nault@uhn.ca).

For any other circulation inquiries:

Toronto General Hospital Library: (416) 340-3429

Toronto Western Hospital Library: (416) 603-5750

Toronto Rehab Library: (416) 597-3422, ext. 3050

Princess Margaret Library: (416) 946-4482

Search strategy saved as 2022-09-26 AB YH Headache OR Craniofacial Pain and Ketamine - Searches

# MEDLINE

Ovid MEDLINE(R) ALL 1946 to September 23, 2022

| **#** | **Searches** | **Results** |
| --- | --- | --- |
| 1 | Bell Palsy/ | 1497 |
| 2 | Eye Pain/ | 783 |
| 3 | Facial Hemiatrophy/ | 985 |
| 4 | exp Facial Neuralgia/ | 10969 |
| 5 | Facial Nerve Diseases/ | 1677 |
| 6 | Facial Nerve Injuries/ | 2124 |
| 7 | Facial Neuralgia/ | 1280 |
| 8 | exp Facial Pain/ | 9619 |
| 9 | Facial Paralysis/ | 13011 |
| 10 | Headache/ | 30500 |
| 11 | exp Headache Disorders/ | 38918 |
| 12 | Hemifacial Spasm/ | 1358 |
| 13 | Herpes Zoster Oticus/ | 530 |
| 14 | Lingual Nerve Injuries/ | 445 |
| 15 | Melkersson-Rosenthal Syndrome/ | 866 |
| 16 | Mobius Syndrome/ | 362 |
| 17 | Myofascial Pain Syndromes/ | 1921 |
| 18 | Neck Pain/ | 8175 |
| 19 | Temporomandibular Joint Dysfunction Syndrome/ | 4933 |
| 20 | Trigeminal Nerve Diseases/ | 1031 |
| 21 | Trigeminal Nerve Injuries/ | 1566 |
| 22 | Trigeminal Neuralgia/ | 7402 |
| 23 | exp Trigeminal Autonomic Cephalalgias/ | 3242 |
| 24 | (bell?? adj2 pals*).mp. | 2896 |
| 25 | (cranial facial* adj2 pain*).mp. | 1 |
| 26 | (craniofacial* adj2 pain*).mp. | 339 |
| 27 | (cranio-facial* adj2 pain*).mp. | 29 |
| 28 | (facial* adj2 neuralg*).mp. | 1445 |
| 29 | (facial* adj2 pain*).mp. | 9748 |
| 30 | (hemifacial adj2 spasm*).mp. | 2230 |
| 31 | (myofascia* adj2 pain*).mp. | 3178 |
| 32 | (orofacial* adj2 neuralg*).mp. | 8 |
| 33 | (oro-facial* adj2 neuralg*).mp. | 0 |
| 34 | (orofacial* adj2 pain*).mp. | 2288 |
| 35 | (oro-facial* adj2 pain*).mp. | 134 |
| 36 | (temporomandibular joint? adj2 d#sfunct*).mp. | 5428 |
| 37 | (temporo-mandibular joint? adj2 d#sfunct*).mp. | 36 |
| 38 | (TMJ adj2 d#sfunct*).mp. | 689 |
| 39 | (trigemina* adj2 nerv*).mp. | 15105 |
| 40 | (trigemina* adj2 neuralgi*).mp. | 8434 |
| 41 | 7th cranial nerve injur*.mp. | 0 |
| 42 | 7th cranial nerve paraly*.mp. | 1 |
| 43 | central facial nerve pals*.mp. | 8 |
| 44 | cephalalgi*.mp. | 913 |
| 45 | cephalgi*.mp. | 407 |
| 46 | earache?.mp. | 1131 |
| 47 | ear-ache?.mp. | 36 |
| 48 | face pain*.mp. | 264 |
| 49 | facial diplegi??.mp. | 313 |
| 50 | facial nerve pals*.mp. | 2021 |
| 51 | facial nerve paralys*.mp. | 1259 |
| 52 | facial nerve pares#s.mp. | 313 |
| 53 | facial nerve trauma*.mp. | 32 |
| 54 | facial neuralgi*.mp. | 1364 |
| 55 | facial pain*.mp. | 9276 |
| 56 | facial pals*.mp. | 4481 |
| 57 | facial paralys*.mp. | 14217 |
| 58 | facial pares#s.mp. | 651 |
| 59 | facialgia??.mp. | 2 |
| 60 | facialis paralys#s.mp. | 2 |
| 61 | glossalgi*.mp. | 304 |
| 62 | headache?.mp,jw. | 97451 |
| 63 | head-ache?.mp,jw. | 90 |
| 64 | heavy-headedness.mp. | 13 |
| 65 | hemicrania continua.mp. | 389 |
| 66 | hemifacial atroph*.mp. | 289 |
| 67 | hemifacial spas*.mp. | 2226 |
| 68 | herpes zoster oticus.mp. | 620 |
| 69 | melkersson rosenthal syndrom*.mp. | 912 |
| 70 | migrain*.mp. | 40578 |
| 71 | moebius syndrome*.mp. | 284 |
| 72 | ophthalmoplegi*.mp. | 11408 |
| 73 | prosopoplegia??.mp. | 4 |
| 74 | seventh cranial nerve injur*.mp. | 5 |
| 75 | seventh cranial nerve paralys#s.mp. | 5 |
| 76 | slit ventricle syndrome*.mp. | 170 |
| 77 | SUNA.mp. | 125 |
| 78 | SUNCT.mp. | 376 |
| 79 | temporal arteritis.mp. | 2183 |
| 80 | toothache?.mp. | 3602 |
| 81 | tooth-ache?.mp. | 38 |
| 82 | trigeminus neuralgi???.mp. | 47 |
| 83 | or/1-82 [ Headache or Facial Pain or Craniofacial Pain & related terms ] | 195422 |
| 84 | exp Neuralgia/ | 23775 |
| 85 | exp Neurons, Afferent/ | 141978 |
| 86 | Nociceptive Pain/ | 954 |
| 87 | exp Nociceptors/ | 11292 |
| 88 | Pain Clinics/ | 1590 |
| 89 | Pain Insensitivity, Congenital/ | 503 |
| 90 | Pain Management/ | 39778 |
| 91 | Pain Measurement/ | 93737 |
| 92 | Pain Perception/ | 3106 |
| 93 | Pain Threshold/ | 14030 |
| 94 | Pain, Intractable/ | 6339 |
| 95 | Pain, Postoperative/ | 46140 |
| 96 | Pain, Procedural/ | 725 |
| 97 | Pain, Referred/ | 373 |
| 98 | Somatoform Disorders/ | 9564 |
| 99 | Somatosensory Disorders/ | 1284 |
| 100 | (ache or aches or ached or aching or achy or achiness).mp. | 19759 |
| 101 | (afferent adj2 neuron?).mp. | 26346 |
| 102 | (deafferentation adj2 pain*).mp. | 309 |
| 103 | (nerve? adj2 injur*).mp. | 36290 |
| 104 | (neuro* adj2 pain*).mp. | 28493 |
| 105 | (neuropath* adj2 pain*).mp. | 23262 |
| 106 | (pain or pains or pained or painful*).mp. | 744240 |
| 107 | (pain* adj3 syndrom*).mp. | 22324 |
| 108 | dysaesthesi*.mp. | 379 |
| 109 | dysesthesi*.mp. | 2026 |
| 110 | Herpes Zoster.mp. | 15273 |
| 111 | hyperalges*.mp. | 19928 |
| 112 | hyperpathi*.mp. | 190 |
| 113 | neuralgi*.mp. | 29710 |
| 114 | neuropathic*.mp. | 29240 |
| 115 | neuropathies.mp. | 33625 |
| 116 | nocicept*.mp. | 40476 |
| 117 | paraesthesi*.mp. | 1993 |
| 118 | paresthesi*.mp. | 12092 |
| 119 | reflex sympathetic dystroph*.mp. | 4081 |
| 120 | shingles.mp. | 1369 |
| 121 | or/84-120 [ Neuropathic pain ] | 997104 |
| 122 | exp Cranial Nerve Injuries/ | 9520 |
| 123 | exp Face/ | 170166 |
| 124 | exp Facial Bones/ | 136329 |
| 125 | exp Facial Injuries/ | 46372 |
| 126 | exp Head/ | 226417 |
| 127 | exp Skull Fractures/ | 22904 |
| 128 | exp Skull/ | 210264 |
| 129 | or/122-128 [ Facial OR Craniofacial Elements ] | 449702 |
| 130 | 121 and 129 | 27709 |
| 131 | 83 or 130 [ Very broad search for Facial or Craniofacial Pain & related terms ] | 213925 |
| 132 | Ketamine/ | 14348 |
| 133 | Receptors, N-Methyl-D-Aspartate/ai | 8807 |
| 134 | 690G0D6V8H.rn. | 14348 |
| 135 | 6740-88-1.rn,rw. | 0 |
| 136 | Anesject??.mp. | 0 |
| 137 | Brevinaze??.mp. | 0 |
| 138 | Cyclohexanes/ and 19680101:19721231.dt. [ Historical ] | 17 |
| 139 | Cyclohexane?.mp. and 19680101:19721231.dt. [ Historical ] | 17 |
| 140 | Cyclohexanone?.mp. | 3573 |
| 141 | "2-(2-chlorophenyl)-2-(methylamino)cyclohexanone".mp. | 2 |
| 142 | "CLSTA 20".mp. | 0 |
| 143 | "ci 581".mp. | 260 |
| 144 | "ci581".mp. | 5 |
| 145 | calipsol??.mp. | 18 |
| 146 | calypsol??.mp. | 37 |
| 147 | dl-ketamine??.mp. | 0 |
| 148 | Esketamine??.mp. | 411 |
| 149 | Etamine??.mp. | 1 |
| 150 | Ivanes??.mp. | 0 |
| 151 | kalipsol??.mp. | 33 |
| 152 | Kanox??.mp. | 0 |
| 153 | Keiran??.mp. | 20 |
| 154 | Keta.mp. | 474 |
| 155 | Keta-Hameln.mp. | 0 |
| 156 | Ketacor??.mp. | 0 |
| 157 | ketaject??.mp. | 2 |
| 158 | ketalar??.mp. | 212 |
| 159 | Ketalin??.mp. | 0 |
| 160 | Ketamax??.mp. | 0 |
| 161 | ketamine??.mp. | 20411 |
| 162 | ketanest??.mp. | 34 |
| 163 | ketaset??.mp. | 14 |
| 164 | Ketava??.mp. | 0 |
| 165 | Ketaved??.mp. | 0 |
| 166 | Ketazol??.mp. | 38 |
| 167 | ketoject??.mp. | 0 |
| 168 | Ketolar??.mp. | 14 |
| 169 | Narkamon??.mp. | 3 |
| 170 | (N-methyl-D-aspartate adj3 receptor inhibit*).mp. | 63 |
| 171 | (NMDA adj2 receptor inhibit*).mp. | 207 |
| 172 | NMDA-antagonis*.mp. | 3991 |
| 173 | Quetanex??.mp. | 0 |
| 174 | S-ketamine??.mp. | 605 |
| 175 | Spravato??.mp. | 21 |
| 176 | Velonarcon??.mp. | 1 |
| 177 | Vetalar??.mp. | 8 |
| 178 | or/132-177 [ Ketamine ] | 34986 |
| 179 | 131 and 178 [ Headache/Facial Pain/Craniofacial Pain + Ketamine ] | 338 |
| 180 | exp animals/ not (exp animals/ and exp humans/) | 5049593 |
| 181 | (animal or animals or ape or apes or baboon or baboons or bonobo or bonobos or cat or cats or chimpanzee or chimpanzees or dog or dogs or feline or felines or ferret or ferrets or flea or fleas or goat or goats or horse or horses or lamb or lambs or macaque or macaques or mandrill or mandrills or mice or mink or minks or monkeys or monkeys or mouse or murine or pig or pigs or porcine or orangutan or orangutans or rat or rats or rodent or rodents or sheep or tamarin or tamarins or veterinary or veterinarian or veterinarians or weasel or weasels or veterinar*).ti. | 2020018 |
| 182 | 180 or 181 | 5336199 |
| 183 | 179 not 182 | 237 |
| 184 | limit 179 to humans | 236 |
| 185 | 183 or 184 | 237 |
| 186 | remove duplicates from 185 | 235 |
| 187 | 202011*:2023*.(da). | 2570239 |
| 188 | 202011*:2023*.(dt). | 1801534 |
| 189 | 202011*:2023*.(ep). | 1402547 |
| 190 | 202011*:2023*.(ez). | 1802314 |
| 191 | or/187-190 [ Update Period ] | 2571066 |
| 192 | 186 and 191 [ Update Citations ] | 52 |

# Medline In-Process

Ovid MEDLINE(R) Epub Ahead of Print and In-Process, In-Data-Review & Other Non-Indexed Citations September 23, 2022

| **#** | **Searches** | **Results** |
| --- | --- | --- |
| 1 | Bell Palsy/ | 0 |
| 2 | Eye Pain/ | 0 |
| 3 | Facial Hemiatrophy/ | 0 |
| 4 | exp Facial Neuralgia/ | 0 |
| 5 | Facial Nerve Diseases/ | 0 |
| 6 | Facial Nerve Injuries/ | 0 |
| 7 | Facial Neuralgia/ | 0 |
| 8 | exp Facial Pain/ | 0 |
| 9 | Facial Paralysis/ | 0 |
| 10 | Headache/ | 0 |
| 11 | exp Headache Disorders/ | 0 |
| 12 | Hemifacial Spasm/ | 0 |
| 13 | Herpes Zoster Oticus/ | 0 |
| 14 | Lingual Nerve Injuries/ | 0 |
| 15 | Melkersson-Rosenthal Syndrome/ | 0 |
| 16 | Mobius Syndrome/ | 0 |
| 17 | Myofascial Pain Syndromes/ | 0 |
| 18 | Neck Pain/ | 0 |
| 19 | Temporomandibular Joint Dysfunction Syndrome/ | 0 |
| 20 | Trigeminal Nerve Diseases/ | 0 |
| 21 | Trigeminal Nerve Injuries/ | 0 |
| 22 | Trigeminal Neuralgia/ | 0 |
| 23 | exp Trigeminal Autonomic Cephalalgias/ | 0 |
| 24 | (bell?? adj2 pals*).mp. | 358 |
| 25 | (cranial facial* adj2 pain*).mp. | 0 |
| 26 | (craniofacial* adj2 pain*).mp. | 56 |
| 27 | (cranio-facial* adj2 pain*).mp. | 2 |
| 28 | (facial* adj2 neuralg*).mp. | 85 |
| 29 | (facial* adj2 pain*).mp. | 709 |
| 30 | (hemifacial adj2 spasm*).mp. | 252 |
| 31 | (myofascia* adj2 pain*).mp. | 418 |
| 32 | (orofacial* adj2 neuralg*).mp. | 2 |
| 33 | (oro-facial* adj2 neuralg*).mp. | 0 |
| 34 | (orofacial* adj2 pain*).mp. | 374 |
| 35 | (oro-facial* adj2 pain*).mp. | 6 |
| 36 | (temporomandibular joint? adj2 d#sfunct*).mp. | 92 |
| 37 | (temporo-mandibular joint? adj2 d#sfunct*).mp. | 2 |
| 38 | (TMJ adj2 d#sfunct*).mp. | 56 |
| 39 | (trigemina* adj2 nerv*).mp. | 946 |
| 40 | (trigemina* adj2 neuralgi*).mp. | 912 |
| 41 | 7th cranial nerve injur*.mp. | 0 |
| 42 | 7th cranial nerve paraly*.mp. | 0 |
| 43 | central facial nerve pals*.mp. | 3 |
| 44 | cephalalgi*.mp. | 102 |
| 45 | cephalgi*.mp. | 48 |
| 46 | earache?.mp. | 89 |
| 47 | ear-ache?.mp. | 14 |
| 48 | face pain*.mp. | 42 |
| 49 | facial diplegi??.mp. | 53 |
| 50 | facial nerve pals*.mp. | 426 |
| 51 | facial nerve paralys*.mp. | 180 |
| 52 | facial nerve pares#s.mp. | 40 |
| 53 | facial nerve trauma*.mp. | 14 |
| 54 | facial neuralgi*.mp. | 68 |
| 55 | facial pain*.mp. | 600 |
| 56 | facial pals*.mp. | 698 |
| 57 | facial paralys*.mp. | 604 |
| 58 | facial pares#s.mp. | 73 |
| 59 | facialgia??.mp. | 0 |
| 60 | facialis paralys#s.mp. | 1 |
| 61 | glossalgi*.mp. | 4 |
| 62 | headache?.mp,jw. | 14141 |
| 63 | head-ache?.mp,jw. | 31 |
| 64 | heavy-headedness.mp. | 0 |
| 65 | hemicrania continua.mp. | 29 |
| 66 | hemifacial atroph*.mp. | 56 |
| 67 | hemifacial spas*.mp. | 252 |
| 68 | herpes zoster oticus.mp. | 23 |
| 69 | melkersson rosenthal syndrom*.mp. | 49 |
| 70 | migrain*.mp. | 4365 |
| 71 | moebius syndrome*.mp. | 26 |
| 72 | ophthalmoplegi*.mp. | 792 |
| 73 | prosopoplegia??.mp. | 1 |
| 74 | seventh cranial nerve injur*.mp. | 1 |
| 75 | seventh cranial nerve paralys#s.mp. | 0 |
| 76 | slit ventricle syndrome*.mp. | 12 |
| 77 | SUNA.mp. | 14 |
| 78 | SUNCT.mp. | 31 |
| 79 | temporal arteritis.mp. | 143 |
| 80 | toothache?.mp. | 267 |
| 81 | tooth-ache?.mp. | 32 |
| 82 | trigeminus neuralgi???.mp. | 1 |
| 83 | or/1-82 [ Headache or Facial Pain or Craniofacial Pain & related terms ] | 21827 |
| 84 | exp Neuralgia/ | 0 |
| 85 | exp Neurons, Afferent/ | 0 |
| 86 | Nociceptive Pain/ | 0 |
| 87 | exp Nociceptors/ | 0 |
| 88 | Pain Clinics/ | 0 |
| 89 | Pain Insensitivity, Congenital/ | 0 |
| 90 | Pain Management/ | 0 |
| 91 | Pain Measurement/ | 0 |
| 92 | Pain Perception/ | 0 |
| 93 | Pain Threshold/ | 0 |
| 94 | Pain, Intractable/ | 0 |
| 95 | Pain, Postoperative/ | 0 |
| 96 | Pain, Procedural/ | 0 |
| 97 | Pain, Referred/ | 0 |
| 98 | Somatoform Disorders/ | 0 |
| 99 | Somatosensory Disorders/ | 0 |
| 100 | (ache or aches or ached or aching or achy or achiness).mp. | 2428 |
| 101 | (afferent adj2 neuron?).mp. | 193 |
| 102 | (deafferentation adj2 pain*).mp. | 21 |
| 103 | (nerve? adj2 injur*).mp. | 4334 |
| 104 | (neuro* adj2 pain*).mp. | 5170 |
| 105 | (neuropath* adj2 pain*).mp. | 4218 |
| 106 | (pain or pains or pained or painful*).mp. | 119871 |
| 107 | (pain* adj3 syndrom*).mp. | 3147 |
| 108 | dysaesthesi*.mp. | 19 |
| 109 | dysesthesi*.mp. | 319 |
| 110 | Herpes Zoster.mp. | 1305 |
| 111 | hyperalges*.mp. | 1512 |
| 112 | hyperpathi*.mp. | 10 |
| 113 | neuralgi*.mp. | 2353 |
| 114 | neuropathic*.mp. | 4877 |
| 115 | neuropathies.mp. | 1445 |
| 116 | nocicept*.mp. | 3115 |
| 117 | paraesthesi*.mp. | 286 |
| 118 | paresthesi*.mp. | 1515 |
| 119 | reflex sympathetic dystroph*.mp. | 112 |
| 120 | shingles.mp. | 262 |
| 121 | or/84-120 [ Neuropathic pain ] | 130625 |
| 122 | exp Cranial Nerve Injuries/ | 0 |
| 123 | exp Face/ | 1 |
| 124 | exp Facial Bones/ | 0 |
| 125 | exp Facial Injuries/ | 0 |
| 126 | exp Head/ | 1 |
| 127 | exp Skull Fractures/ | 0 |
| 128 | exp Skull/ | 0 |
| 129 | or/122-128 [ Facial OR Craniofacial Elements ] | 1 |
| 130 | 121 and 129 | 0 |
| 131 | 83 or 130 [ Very broad search for Facial or Craniofacial Pain & related terms ] | 21827 |
| 132 | Ketamine/ | 0 |
| 133 | Receptors, N-Methyl-D-Aspartate/ai | 0 |
| 134 | 690G0D6V8H.rn. | 0 |
| 135 | 6740-88-1.rn,rw. | 0 |
| 136 | Anesject??.mp. | 0 |
| 137 | Brevinaze??.mp. | 0 |
| 138 | Cyclohexanes/ and 19680101:19721231.dt. [ Historical ] | 0 |
| 139 | Cyclohexane?.mp. and 19680101:19721231.dt. [ Historical ] | 0 |
| 140 | Cyclohexanone?.mp. | 669 |
| 141 | "2-(2-chlorophenyl)-2-(methylamino)cyclohexanone".mp. | 1 |
| 142 | "CLSTA 20".mp. | 1 |
| 143 | "ci 581".mp. | 19 |
| 144 | "ci581".mp. | 1 |
| 145 | calipsol??.mp. | 0 |
| 146 | calypsol??.mp. | 0 |
| 147 | dl-ketamine??.mp. | 2 |
| 148 | Esketamine??.mp. | 120 |
| 149 | Etamine??.mp. | 2 |
| 150 | Ivanes??.mp. | 0 |
| 151 | kalipsol??.mp. | 0 |
| 152 | Kanox??.mp. | 0 |
| 153 | Keiran??.mp. | 1 |
| 154 | Keta.mp. | 55 |
| 155 | Keta-Hameln.mp. | 0 |
| 156 | Ketacor??.mp. | 0 |
| 157 | ketaject??.mp. | 1 |
| 158 | ketalar??.mp. | 3 |
| 159 | Ketalin??.mp. | 0 |
| 160 | Ketamax??.mp. | 0 |
| 161 | ketamine??.mp. | 2497 |
| 162 | ketanest??.mp. | 1 |
| 163 | ketaset??.mp. | 2 |
| 164 | Ketava??.mp. | 1 |
| 165 | Ketaved??.mp. | 0 |
| 166 | Ketazol??.mp. | 1 |
| 167 | ketoject??.mp. | 0 |
| 168 | Ketolar??.mp. | 0 |
| 169 | Narkamon??.mp. | 0 |
| 170 | (N-methyl-D-aspartate adj3 receptor inhibit*).mp. | 5 |
| 171 | (NMDA adj2 receptor inhibit*).mp. | 19 |
| 172 | NMDA-antagonis*.mp. | 208 |
| 173 | Quetanex??.mp. | 0 |
| 174 | S-ketamine??.mp. | 86 |
| 175 | Spravato??.mp. | 8 |
| 176 | Velonarcon??.mp. | 0 |
| 177 | Vetalar??.mp. | 1 |
| 178 | or/132-177 [ Ketamine ] | 3460 |
| 179 | 131 and 178 [ Headache/Facial Pain/Craniofacial Pain + Ketamine ] | 31 |
| 180 | (animal or animals or ape or apes or baboon or baboons or bonobo or bonobos or cat or cats or chimpanzee or chimpanzees or dog or dogs or feline or felines or ferret or ferrets or flea or fleas or goat or goats or horse or horses or lamb or lambs or macaque or macaques or mandrill or mandrills or mice or mink or minks or monkeys or monkeys or mouse or murine or pig or pigs or porcine or orangutan or orangutans or rat or rats or rodent or rodents or sheep or tamarin or tamarins or veterinary or veterinarian or veterinarians or weasel or weasels or veterinar*).ti. | 128577 |
| 181 | 179 not 180 | 30 |
| 182 | remove duplicates from 181 | 30 |
| 183 | 202011*:2023*.(da). | 1119153 |
| 184 | 202011*:2023*.(dt). | 1201732 |
| 185 | 202011*:2023*.(ep). | 973590 |
| 186 | 202011*:2023*.(ez). | 1119064 |
| 187 | or/183-186 [ Update Period ] | 1205149 |
| 188 | 182 and 187 [ Update Citations ] | 14 |

# Embase

Embase Classic+Embase 1947 to 2022 September 23

| **#** | **Searches** | **Results** |
| --- | --- | --- |
| 1 | Bell Palsy/ | 4709 |
| 2 | Eye Pain/ | 11613 |
| 3 | Facial Hemiatrophy/ | 1346 |
| 4 | exp Facial Neuralgia/ | 12506 |
| 5 | Facial Nerve Diseases/ | 1292 |
| 6 | Facial Nerve Injuries/ | 1375 |
| 7 | Facial Neuralgia/ | 11296 |
| 8 | exp Facial Pain/ | 12506 |
| 9 | Facial Paralysis/ | 16057 |
| 10 | Headache/ | 264314 |
| 11 | exp Headache Disorders/ | 369206 |
| 12 | Hemifacial Spasm/ | 3418 |
| 13 | Herpes Zoster Oticus/ | 1400 |
| 14 | Lingual Nerve Injuries/ | 158 |
| 15 | Melkersson-Rosenthal Syndrome/ | 1066 |
| 16 | Mobius Syndrome/ | 946 |
| 17 | Myofascial Pain Syndromes/ | 7544 |
| 18 | Neck Pain/ | 28849 |
| 19 | Temporomandibular Joint Dysfunction Syndrome/ | 13390 |
| 20 | Trigeminal Nerve Diseases/ | 1028 |
| 21 | Trigeminal Nerve Injuries/ | 352 |
| 22 | Trigeminal Neuralgia/ | 7355 |
| 23 | exp "headache and facial pain"/ [ Embase ] | 369206 |
| 24 | facial nerve injury/ | 2264 |
| 25 | exp facial nerve paralysis/ | 33886 |
| 26 | hemifacial atrophy/ | 1741 |
| 27 | hemifacial spasm/ | 3418 |
| 28 | herpes zoster oticus/ | 1400 |
| 29 | melkersson rosenthal syndrome/ | 1066 |
| 30 | moebius syndrome/ | 1047 |
| 31 | exp Trigeminal Autonomic Cephalalgias/ | 7420 |
| 32 | trigeminus neuralgia/ | 13473 |
| 33 | (bell?? adj2 pals*).mp. | 5796 |
| 34 | (cranial facial* adj2 pain*).mp. | 5 |
| 35 | (craniofacial* adj2 pain*).mp. | 541 |
| 36 | (cranio-facial* adj2 pain*).mp. | 49 |
| 37 | (facial* adj2 neuralg*).mp. | 721 |
| 38 | (facial* adj2 pain*).mp. | 9149 |
| 39 | (hemifacial adj2 spasm*).mp. | 3864 |
| 40 | (myofascia* adj2 pain*).mp. | 9611 |
| 41 | (orofacial* adj2 neuralg*).mp. | 10 |
| 42 | (oro-facial* adj2 neuralg*).mp. | 0 |
| 43 | (orofacial* adj2 pain*).mp. | 3258 |
| 44 | (oro-facial* adj2 pain*).mp. | 158 |
| 45 | (temporomandibular joint? adj2 d#sfunct*).mp. | 1427 |
| 46 | (temporo-mandibular joint? adj2 d#sfunct*).mp. | 62 |
| 47 | (TMJ adj2 d#sfunct*).mp. | 859 |
| 48 | (trigemina* adj2 nerv*).mp. | 20327 |
| 49 | (trigemina* adj2 neuralgi*).mp. | 9912 |
| 50 | 7th cranial nerve injur*.mp. | 0 |
| 51 | 7th cranial nerve paraly*.mp. | 5 |
| 52 | central facial nerve pals*.mp. | 23 |
| 53 | cephalalgi*.mp. | 2280 |
| 54 | cephalgi*.mp. | 840 |
| 55 | earache?.mp. | 801 |
| 56 | ear-ache?.mp. | 95 |
| 57 | face pain*.mp. | 12746 |
| 58 | facial diplegi??.mp. | 590 |
| 59 | facial nerve pals*.mp. | 3404 |
| 60 | facial nerve paralys*.mp. | 29195 |
| 61 | facial nerve pares#s.mp. | 513 |
| 62 | facial nerve trauma*.mp. | 53 |
| 63 | facial neuralgi*.mp. | 511 |
| 64 | facial pain*.mp. | 8226 |
| 65 | facial pals*.mp. | 7710 |
| 66 | facial paralys*.mp. | 8108 |
| 67 | facial pares#s.mp. | 1300 |
| 68 | facialgia??.mp. | 6 |
| 69 | facialis paralys#s.mp. | 9 |
| 70 | glossalgi*.mp. | 97 |
| 71 | headache?.mp,jw. | 340992 |
| 72 | head-ache?.mp,jw. | 361 |
| 73 | heavy-headedness.mp. | 55 |
| 74 | hemicrania continua.mp. | 836 |
| 75 | hemifacial atroph*.mp. | 1773 |
| 76 | hemifacial spas*.mp. | 3858 |
| 77 | herpes zoster oticus.mp. | 1476 |
| 78 | melkersson rosenthal syndrom*.mp. | 1187 |
| 79 | migrain*.mp. | 86046 |
| 80 | moebius syndrome*.mp. | 1136 |
| 81 | ophthalmoplegi*.mp. | 20891 |
| 82 | prosopoplegia??.mp. | 9 |
| 83 | seventh cranial nerve injur*.mp. | 7 |
| 84 | seventh cranial nerve paralys#s.mp. | 5 |
| 85 | slit ventricle syndrome*.mp. | 284 |
| 86 | SUNA.mp. | 241 |
| 87 | SUNCT.mp. | 755 |
| 88 | temporal arteritis.mp. | 5811 |
| 89 | toothache?.mp. | 2194 |
| 90 | tooth-ache?.mp. | 109 |
| 91 | trigeminus neuralgi???.mp. | 13477 |
| 92 | or/1-91 [ Headache or Facial Pain or Craniofacial Pain & related terms ] | 522328 |
| 93 | Ketamine/ | 52627 |
| 94 | 690G0D6V8H.rn. | 0 |
| 95 | 6740-88-1.rn,rw. | 48850 |
| 96 | Anesject??.mp. | 0 |
| 97 | Brevinaze??.mp. | 0 |
| 98 | Cyclohexanone?.mp. | 4952 |
| 99 | "2-(2-chlorophenyl)-2-(methylamino)cyclohexanone".mp. | 7 |
| 100 | "CLSTA 20".mp. | 0 |
| 101 | "ci 581".mp. | 458 |
| 102 | "ci581".mp. | 8 |
| 103 | calipsol??.mp. | 34 |
| 104 | calypsol??.mp. | 67 |
| 105 | dl-ketamine??.mp. | 3 |
| 106 | Etamine??.mp. | 24 |
| 107 | Esketamine??.mp. | 1228 |
| 108 | Ivanes??.mp. | 11 |
| 109 | kalipsol??.mp. | 27 |
| 110 | Kanox??.mp. | 0 |
| 111 | Keiran??.mp. | 23 |
| 112 | Keta.mp. | 573 |
| 113 | Keta-Hameln.mp. | 0 |
| 114 | Ketacor??.mp. | 0 |
| 115 | ketaject??.mp. | 72 |
| 116 | ketalar??.mp. | 1600 |
| 117 | Ketalin??.mp. | 5 |
| 118 | Ketamax??.mp. | 5 |
| 119 | ketamine??.mp. | 56792 |
| 120 | ketanest??.mp. | 406 |
| 121 | ketaset??.mp. | 328 |
| 122 | Ketava??.mp. | 2 |
| 123 | Ketaved??.mp. | 20 |
| 124 | Ketazol??.mp. | 354 |
| 125 | ketoject??.mp. | 2 |
| 126 | Ketolar??.mp. | 79 |
| 127 | Narkamon??.mp. | 24 |
| 128 | (N-methyl-D-aspartate adj3 receptor inhibit*).mp. | 75 |
| 129 | (NMDA adj2 receptor inhibit*).mp. | 282 |
| 130 | NMDA-antagonis*.mp. | 5404 |
| 131 | Quetanex??.mp. | 0 |
| 132 | S-ketamine??.mp. | 1713 |
| 133 | Spravato??.mp. | 67 |
| 134 | Velonarcon??.mp. | 8 |
| 135 | Vetalar??.mp. | 185 |
| 136 | or/93-135 [ Ketamine & related terms ] | 68058 |
| 137 | 92 and 136 [ Headache/Facial Pain/Craniofacial Pain + Ketamine ] | 1835 |
| 138 | (exp animals/ or exp animal experimentation/ or nonhuman/) not ((exp animals/ or exp animal experimentation/ or nonhuman/) and exp human/) | 7786315 |
| 139 | (animal or animals or ape or apes or baboon or baboons or bonobo or bonobos or cat or cats or chimpanzee or chimpanzees or dog or dogs or feline or felines or ferret or ferrets or flea or fleas or goat or goats or horse or horses or lamb or lambs or macaque or macaques or mandrill or mandrills or mice or mink or minks or monkeys or monkeys or mouse or murine or pig or pigs or porcine or orangutan or orangutans or rat or rats or rodent or rodents or sheep or tamarin or tamarins or veterinary or veterinarian or veterinarians or weasel or weasels or veterinar*).ti. | 2625388 |
| 140 | 138 or 139 | 8132319 |
| 141 | 137 not 140 | 1678 |
| 142 | limit 137 to human | 1646 |
| 143 | 141 or 142 | 1693 |
| 144 | remove duplicates from 143 | 1684 |
| 145 | limit 144 to dc=20201101-20231231 [ Update Period / Update Citations ] | 314 |

# CCTR

EBM Reviews - Cochrane Central Register of Controlled Trials August 2022

| **#** | **Searches** | **Results** |
| --- | --- | --- |
| 1 | Bell Palsy/ | 117 |
| 2 | Eye Pain/ | 121 |
| 3 | Facial Hemiatrophy/ | 4 |
| 4 | exp Facial Neuralgia/ | 279 |
| 5 | Facial Nerve Diseases/ | 21 |
| 6 | Facial Nerve Injuries/ | 23 |
| 7 | Facial Neuralgia/ | 36 |
| 8 | exp Facial Pain/ | 807 |
| 9 | Facial Paralysis/ | 179 |
| 10 | Headache/ | 2612 |
| 11 | exp Headache Disorders/ | 3817 |
| 12 | Hemifacial Spasm/ | 47 |
| 13 | Herpes Zoster Oticus/ | 6 |
| 14 | Lingual Nerve Injuries/ | 16 |
| 15 | Melkersson-Rosenthal Syndrome/ | 3 |
| 16 | Mobius Syndrome/ | 1 |
| 17 | Myofascial Pain Syndromes/ | 1042 |
| 18 | Neck Pain/ | 1551 |
| 19 | Temporomandibular Joint Dysfunction Syndrome/ | 359 |
| 20 | Trigeminal Nerve Diseases/ | 12 |
| 21 | Trigeminal Nerve Injuries/ | 53 |
| 22 | Trigeminal Neuralgia/ | 176 |
| 23 | exp "headache and facial pain"/ [ Embase ] | 0 |
| 24 | facial nerve injury/ | 23 |
| 25 | exp facial nerve paralysis/ | 0 |
| 26 | hemifacial atrophy/ | 4 |
| 27 | hemifacial spasm/ | 47 |
| 28 | herpes zoster oticus/ | 6 |
| 29 | melkersson rosenthal syndrome/ | 3 |
| 30 | moebius syndrome/ | 1 |
| 31 | exp Trigeminal Autonomic Cephalalgias/ | 141 |
| 32 | trigeminus neuralgia/ | 0 |
| 33 | (bell?? adj2 pals*).mp. | 362 |
| 34 | (cranial facial* adj2 pain*).mp. | 2 |
| 35 | (craniofacial* adj2 pain*).mp. | 39 |
| 36 | (cranio-facial* adj2 pain*).mp. | 4 |
| 37 | (facial* adj2 neuralg*).mp. | 59 |
| 38 | (facial* adj2 pain*).mp. | 1372 |
| 39 | (hemifacial adj2 spasm*).mp. | 147 |
| 40 | (myofascia* adj2 pain*).mp. | 2100 |
| 41 | (orofacial* adj2 neuralg*).mp. | 0 |
| 42 | (oro-facial* adj2 neuralg*).mp. | 1 |
| 43 | (orofacial* adj2 pain*).mp. | 244 |
| 44 | (oro-facial* adj2 pain*).mp. | 19 |
| 45 | (temporomandibular joint? adj2 d#sfunct*).mp. | 461 |
| 46 | (temporo-mandibular joint? adj2 d#sfunct*).mp. | 7 |
| 47 | (TMJ adj2 d#sfunct*).mp. | 50 |
| 48 | (trigemina* adj2 nerv*).mp. | 489 |
| 49 | (trigemina* adj2 neuralgi*).mp. | 482 |
| 50 | 7th cranial nerve injur*.mp. | 0 |
| 51 | 7th cranial nerve paraly*.mp. | 0 |
| 52 | central facial nerve pals*.mp. | 0 |
| 53 | cephalalgi*.mp. | 69 |
| 54 | cephalgi*.mp. | 30 |
| 55 | earache?.mp. | 105 |
| 56 | ear-ache?.mp. | 3 |
| 57 | face pain*.mp. | 368 |
| 58 | facial diplegi??.mp. | 0 |
| 59 | facial nerve pals*.mp. | 77 |
| 60 | facial nerve paralys*.mp. | 257 |
| 61 | facial nerve pares#s.mp. | 20 |
| 62 | facial nerve trauma*.mp. | 1 |
| 63 | facial neuralgi*.mp. | 39 |
| 64 | facial pain*.mp. | 1091 |
| 65 | facial pals*.mp. | 211 |
| 66 | facial paralys*.mp. | 480 |
| 67 | facial pares#s.mp. | 31 |
| 68 | facialgia??.mp. | 0 |
| 69 | facialis paralys#s.mp. | 0 |
| 70 | glossalgi*.mp. | 7 |
| 71 | headache?.mp,jw. | 37498 |
| 72 | head-ache?.mp,jw. | 33 |
| 73 | heavy-headedness.mp. | 11 |
| 74 | hemicrania continua.mp. | 16 |
| 75 | hemifacial atroph*.mp. | 7 |
| 76 | hemifacial spas*.mp. | 147 |
| 77 | herpes zoster oticus.mp. | 17 |
| 78 | melkersson rosenthal syndrom*.mp. | 3 |
| 79 | migrain*.mp. | 9438 |
| 80 | moebius syndrome*.mp. | 2 |
| 81 | ophthalmoplegi*.mp. | 102 |
| 82 | prosopoplegia??.mp. | 1 |
| 83 | seventh cranial nerve injur*.mp. | 0 |
| 84 | seventh cranial nerve paralys#s.mp. | 0 |
| 85 | slit ventricle syndrome*.mp. | 2 |
| 86 | SUNA.mp. | 4 |
| 87 | SUNCT.mp. | 5 |
| 88 | temporal arteritis.mp. | 44 |
| 89 | toothache?.mp. | 364 |
| 90 | tooth-ache?.mp. | 5 |
| 91 | trigeminus neuralgi???.mp. | 183 |
| 92 | or/1-91 [ Headache or Facial Pain or Craniofacial Pain & related terms ] | 48118 |
| 93 | Ketamine/ | 2506 |
| 94 | [690G0D6V8H.rn.] | 0 |
| 95 | [6740-88-1.rn,rw.] | 0 |
| 96 | Anesject??.mp. | 0 |
| 97 | Brevinaze??.mp. | 0 |
| 98 | Cyclohexanone?.mp. | 22 |
| 99 | "2-(2-chlorophenyl)-2-(methylamino)cyclohexanone".mp. | 2 |
| 100 | "CLSTA 20".mp. | 0 |
| 101 | "ci 581".mp. | 37 |
| 102 | "ci581".mp. | 0 |
| 103 | calipsol??.mp. | 0 |
| 104 | calypsol??.mp. | 1 |
| 105 | dl-ketamine??.mp. | 1 |
| 106 | Etamine??.mp. | 1 |
| 107 | Esketamine??.mp. | 488 |
| 108 | Ivanes??.mp. | 6 |
| 109 | kalipsol??.mp. | 1 |
| 110 | Kanox??.mp. | 0 |
| 111 | Keiran??.mp. | 0 |
| 112 | Keta.mp. | 16 |
| 113 | Keta-Hameln.mp. | 0 |
| 114 | Ketacor??.mp. | 0 |
| 115 | ketaject??.mp. | 0 |
| 116 | ketalar??.mp. | 57 |
| 117 | Ketalin??.mp. | 0 |
| 118 | Ketamax??.mp. | 0 |
| 119 | ketamine??.mp. | 6264 |
| 120 | ketanest??.mp. | 60 |
| 121 | ketaset??.mp. | 0 |
| 122 | Ketava??.mp. | 0 |
| 123 | Ketaved??.mp. | 0 |
| 124 | Ketazol??.mp. | 40 |
| 125 | ketoject??.mp. | 0 |
| 126 | Ketolar??.mp. | 6 |
| 127 | Narkamon??.mp. | 0 |
| 128 | (N-methyl-D-aspartate adj3 receptor inhibit*).mp. | 6 |
| 129 | (NMDA adj2 receptor inhibit*).mp. | 5 |
| 130 | NMDA-antagonis*.mp. | 335 |
| 131 | Quetanex??.mp. | 0 |
| 132 | S-ketamine??.mp. | 369 |
| 133 | Spravato??.mp. | 8 |
| 134 | Velonarcon??.mp. | 0 |
| 135 | Vetalar??.mp. | 0 |
| 136 | or/93-135 [ Ketamine & related terms ] | 6854 |
| 137 | 92 and 136 [ Headache/Facial Pain/Craniofacial Pain + Ketamine ] | 243 |
| 138 | remove duplicates from 137 | 236 |
| 139 | (animal or animals or ape or apes or baboon or baboons or bonobo or bonobos or cat or cats or chimpanzee or chimpanzees or dog or dogs or feline or felines or ferret or ferrets or flea or fleas or goat or goats or horse or horses or lamb or lambs or macaque or macaques or mandrill or mandrills or mice or mink or minks or monkeys or monkeys or mouse or murine or pig or pigs or porcine or orangutan or orangutans or rat or rats or rodent or rodents or sheep or tamarin or tamarins or veterinary or veterinarian or veterinarians or weasel or weasels or veterinar*).ti. | 4654 |
| 140 | 138 not 139 | 236 |
| 141 | limit 140 to yr="2020 -Current" | 52 |
| 142 | 2020*.dl. | 176457 |
| 143 | 2021*.dl. | 123665 |
| 144 | 2022*.dl. | 96562 |
| 145 | or/142-144 [ Update Period ] | 396684 |
| 146 | 140 and 145 | 67 |
| 147 | 141 or 146 [ Update Citations / Clinical Trials ] | 68 |

# CDSR

EBM Reviews - Cochrane Database of Systematic Reviews 2005 to September 21, 2022

| **#** | **Searches** | **Results** |
| --- | --- | --- |
| 1 | (bell?? adj2 pals*).ti,ab. | 7 |
| 2 | (cranial facial* adj2 pain*).ti,ab. | 0 |
| 3 | (craniofacial* adj2 pain*).ti,ab. | 0 |
| 4 | (cranio-facial* adj2 pain*).ti,ab. | 0 |
| 5 | (facial* adj2 neuralg*).ti,ab. | 0 |
| 6 | (facial* adj2 pain*).ti,ab. | 10 |
| 7 | (hemifacial adj2 spasm*).ti,ab. | 2 |
| 8 | (myofascia* adj2 pain*).ti,ab. | 5 |
| 9 | (orofacial* adj2 neuralg*).ti,ab. | 0 |
| 10 | (oro-facial* adj2 neuralg*).ti,ab. | 0 |
| 11 | (orofacial* adj2 pain*).ti,ab. | 1 |
| 12 | (oro-facial* adj2 pain*).ti,ab. | 0 |
| 13 | (temporomandibular joint? adj2 d#sfunct*).ti,ab. | 0 |
| 14 | (temporo-mandibular joint? adj2 d#sfunct*).ti,ab. | 0 |
| 15 | (TMJ adj2 d#sfunct*).ti,ab. | 0 |
| 16 | (trigemina* adj2 nerv*).ti,ab. | 4 |
| 17 | (trigemina* adj2 neuralgi*).ti,ab. | 9 |
| 18 | 7th cranial nerve injur*.ti,ab. | 0 |
| 19 | 7th cranial nerve paraly*.ti,ab. | 0 |
| 20 | central facial nerve pals*.ti,ab. | 0 |
| 21 | cephalalgi*.ti,ab. | 4 |
| 22 | cephalgi*.ti,ab. | 0 |
| 23 | earache?.ti,ab. | 0 |
| 24 | ear-ache?.ti,ab. | 0 |
| 25 | face pain*.ti,ab. | 0 |
| 26 | facial diplegi??.ti,ab. | 0 |
| 27 | facial nerve pals*.ti,ab. | 0 |
| 28 | facial nerve paralys*.ti,ab. | 0 |
| 29 | facial nerve pares#s.ti,ab. | 0 |
| 30 | facial nerve trauma*.ti,ab. | 0 |
| 31 | facial neuralgi*.ti,ab. | 0 |
| 32 | facial pain*.ti,ab. | 4 |
| 33 | facial pals*.ti,ab. | 6 |
| 34 | facial paralys*.ti,ab. | 7 |
| 35 | facial pares#s.ti,ab. | 0 |
| 36 | facialgia??.ti,ab. | 0 |
| 37 | facialis paralys#s.ti,ab. | 0 |
| 38 | glossalgi*.ti,ab. | 0 |
| 39 | headache?.ti,ab. | 250 |
| 40 | head-ache?.ti,ab. | 0 |
| 41 | heavy-headedness.ti,ab. | 0 |
| 42 | hemicrania continua.ti,ab. | 0 |
| 43 | hemifacial atroph*.ti,ab. | 0 |
| 44 | hemifacial spas*.ti,ab. | 2 |
| 45 | herpes zoster oticus.ti,ab. | 2 |
| 46 | melkersson rosenthal syndrom*.ti,ab. | 0 |
| 47 | migrain*.ti,ab. | 71 |
| 48 | moebius syndrome*.ti,ab. | 0 |
| 49 | ophthalmoplegi*.ti,ab. | 1 |
| 50 | prosopoplegia??.ti,ab. | 0 |
| 51 | seventh cranial nerve injur*.ti,ab. | 0 |
| 52 | seventh cranial nerve paralys#s.ti,ab. | 0 |
| 53 | slit ventricle syndrome*.ti,ab. | 0 |
| 54 | SUNA.ti,ab. | 0 |
| 55 | SUNCT.ti,ab. | 0 |
| 56 | temporal arteritis.ti,ab. | 0 |
| 57 | toothache?.ti,ab. | 0 |
| 58 | tooth-ache?.ti,ab. | 0 |
| 59 | trigeminus neuralgi???.ti,ab. | 0 |
| 60 | or/1-59 [ Headache / Facial Pain / Craniofacial Pain ] | 310 |
| 61 | Anesject??.ti,ab. | 0 |
| 62 | Brevinaze??.ti,ab. | 0 |
| 63 | Cyclohexanone?.ti,ab. | 0 |
| 64 | "2-(2-chlorophenyl)-2-(methylamino)cyclohexanone".ti,ab. | 0 |
| 65 | "CLSTA 20".ti,ab. | 0 |
| 66 | "ci 581".ti,ab. | 1 |
| 67 | "ci581".ti,ab. | 0 |
| 68 | calipsol??.ti,ab. | 0 |
| 69 | calypsol??.ti,ab. | 0 |
| 70 | dl-ketamine??.ti,ab. | 0 |
| 71 | Etamine??.ti,ab. | 0 |
| 72 | Esketamine??.ti,ab. | 2 |
| 73 | Ivanes??.ti,ab. | 0 |
| 74 | kalipsol??.ti,ab. | 0 |
| 75 | Kanox??.ti,ab. | 0 |
| 76 | Keiran??.ti,ab. | 0 |
| 77 | Keta.ti,ab. | 0 |
| 78 | Keta-Hameln.ti,ab. | 0 |
| 79 | Ketacor??.ti,ab. | 0 |
| 80 | ketaject??.ti,ab. | 0 |
| 81 | ketalar??.ti,ab. | 0 |
| 82 | Ketalin??.ti,ab. | 0 |
| 83 | Ketamax??.ti,ab. | 0 |
| 84 | ketamine??.ti,ab. | 17 |
| 85 | ketanest??.ti,ab. | 0 |
| 86 | ketaset??.ti,ab. | 0 |
| 87 | Ketava??.ti,ab. | 0 |
| 88 | Ketaved??.ti,ab. | 0 |
| 89 | Ketazol??.ti,ab. | 1 |
| 90 | ketoject??.ti,ab. | 0 |
| 91 | Ketolar??.ti,ab. | 0 |
| 92 | Narkamon??.ti,ab. | 0 |
| 93 | (N-methyl-D-aspartate adj3 receptor inhibit*).ti,ab. | 0 |
| 94 | (NMDA adj2 receptor inhibit*).ti,ab. | 0 |
| 95 | NMDA-antagonis*.ti,ab. | 2 |
| 96 | Quetanex??.ti,ab. | 0 |
| 97 | S-ketamine??.ti,ab. | 1 |
| 98 | Spravato??.ti,ab. | 0 |
| 99 | Velonarcon??.ti,ab. | 0 |
| 100 | Vetalar??.ti,ab. | 0 |
| 101 | or/61-100 [ Ketamine ] | 22 |
| 102 | 60 and 101 | 1 |

# Scopus

# 71 document results

( ( ( ( INDEXTERMS ( "Bell Palsy" )  OR  INDEXTERMS ( "Eye Pain" )  OR  INDEXTERMS ( "Facial Hemiatrophy" )  OR  INDEXTERMS ( "Facial Neuralgia" )  OR  INDEXTERMS ( "Facial Nerve Diseases" )  OR  INDEXTERMS ( "Facial Nerve Injuries" )  OR  INDEXTERMS ( "Facial Neuralgia" )  OR  INDEXTERMS ( "Facial Pain" )  OR  INDEXTERMS ( "Facial Paralysis" )  OR  INDEXTERMS ( "Headache" )  OR  INDEXTERMS ( "Headache Disorders" )  OR  INDEXTERMS ( "Hemifacial Spasm" )  OR  INDEXTERMS ( "Herpes Zoster Oticus" )  OR  INDEXTERMS ( "Lingual Nerve Injuries" )  OR  INDEXTERMS ( "Melkersson-Rosenthal Syndrome" )  OR  INDEXTERMS ( "Mobius Syndrome" )  OR  INDEXTERMS ( "Myofascial Pain Syndrome*" )  OR  INDEXTERMS ( "Neck Pain*" )  OR  INDEXTERMS ( "Temporomandibular Joint Dysfunction Syndrome" )  OR  INDEXTERMS ( "Trigeminal Nerve Diseases" )  OR  INDEXTERMS ( "Trigeminal Nerve Injuries" )  OR  INDEXTERMS ( "Trigeminal Neuralgia" )  OR  INDEXTERMS ( "headache and facial pain" )  OR  INDEXTERMS ( "facial nerve injur*" )  OR  INDEXTERMS ( "facial nerve paralysis" )  OR  INDEXTERMS ( "hemifacial atroph*" )  OR  INDEXTERMS ( "hemifacial spasm*" )  OR  INDEXTERMS ( "herpes zoster oticus" )  OR  INDEXTERMS ( "melkersson rosenthal syndrome" )  OR  INDEXTERMS ( "moebius syndrome" )  OR  INDEXTERMS ( "trigeminal autonomic cephalalgia" )  OR  INDEXTERMS ( "trigeminus neuralgia" )  OR  ( "bell*"  W/2  "pals*" )  OR  ( "cranial facial*"  W/2  "pain*" )  OR  ( "craniofacial*"  W/2  "pain*" )  OR  ( "cranio-facial*"  W/2  "pain*" )  OR  ( "facial*"  W/2  "neuralg*" )  OR  ( "facial*"  W/2  "pain*" )  OR  ( "hemifacial"  W/2  "spasm*" )  OR  ( "myofascia*"  W/2  "pain*" )  OR  ( "orofacial*"  W/2  "neuralg*" )  OR  ( "oro-facial*"  W/2  "neuralg*" )  OR  ( "orofacial*"  W/2  "pain*" )  OR  ( "oro-facial*"  W/2  "pain*" )  OR  ( "temporomandibular joint*sfunct*" )  OR  ( "temporo-mandibular joint*sfunct*" )  OR  ( "TMJ"  W/2  "d*sfunct*" )  OR  ( "trigemina*"  W/2  "nerv*" )  OR  ( "trigemina*"  W/2  "neuralgi*" )  OR  TITLE-ABS-KEY ( "7th cranial nerve injur*" )  OR  TITLE-ABS-KEY ( "7th cranial nerve paraly*" )  OR  TITLE-ABS-KEY ( "central facial nerve pals*" )  OR  TITLE-ABS-KEY ( "cephalalgi*" )  OR  TITLE-ABS-KEY ( "cephalgi*" )  OR  TITLE-ABS-KEY ( "earache*" )  OR  TITLE-ABS-KEY ( "ear-ache*" )  OR  TITLE-ABS-KEY ( "face pain*" )  OR  TITLE-ABS-KEY ( "facial diplegi*" )  OR  "facial nerve injur*"  OR  TITLE-ABS-KEY ( "facial nerve pals*" )  OR  TITLE-ABS-KEY ( "facial nerve paralys*" )  OR  "facial nerve pares*"  OR  TITLE-ABS-KEY ( "facial nerve trauma*" )  OR  TITLE-ABS-KEY ( "facial neuralgi*" )  OR  TITLE-ABS-KEY ( "facial pain*" )  OR  TITLE-ABS-KEY ( "facial pals*" )  OR  TITLE-ABS-KEY ( "facial paralys*" )  OR  "facial pares*"  OR  TITLE-ABS-KEY ( "facialgia*" )  OR  "facialis paralys*"  OR  TITLE-ABS-KEY ( "glossalgi*" )  OR  TITLE-ABS-KEY ( "headache*" )  OR  TITLE-ABS-KEY ( "head-ache*" )  OR  TITLE-ABS-KEY ( "heavy-headedness" )  OR  TITLE-ABS-KEY ( "hemicrania continua" )  OR  TITLE-ABS-KEY ( "hemifacial atroph*" )  OR  TITLE-ABS-KEY ( "hemifacial spas*" )  OR  TITLE-ABS-KEY ( "herpes zoster oticus" )  OR  TITLE-ABS-KEY ( "melkersson rosenthal syndrom*" )  OR  TITLE-ABS-KEY ( "migrain*" )  OR  TITLE-ABS-KEY ( "moebius syndrome*" )  OR  TITLE-ABS-KEY ( "ophthalmoplegi*" )  OR  TITLE-ABS-KEY ( "prosopoplegia*" )  OR  TITLE-ABS-KEY ( "seventh cranial nerve injur*" )  OR  "seventh cranial nerve paralys*"  OR  TITLE-ABS-KEY ( "slit ventricle syndrome*" )  OR  TITLE-ABS-KEY ( "SUNA" )  OR  TITLE-ABS-KEY ( "SUNCT" )  OR  TITLE-ABS-KEY ( "temporal arteritis" )  OR  TITLE-ABS-KEY ( "toothache*" )  OR  TITLE-ABS-KEY ( "tooth-ache*" )  OR  TITLE-ABS-KEY ( "trigeminus neuralgi*" ) ) )  AND  ( ( INDEXTERMS ( "Ketamine" )  OR  "690G0D6V8H"  OR  "6740-88-1"  OR  TITLE-ABS-KEY ( "Anesject*" )  OR  TITLE-ABS-KEY ( "Brevinaze*" )  OR  TITLE-ABS-KEY ( "Cyclohexanone*" )  OR  TITLE-ABS-KEY ( "2-(2-chlorophenyl)-2-(methylamino)cyclohexanone" )  OR  TITLE-ABS-KEY ( "CLSTA 20" )  OR  TITLE-ABS-KEY ( "ci 581" )  OR  TITLE-ABS-KEY ( "ci581" )  OR  TITLE-ABS-KEY ( "calipsol*" )  OR  TITLE-ABS-KEY ( "calypsol*" )  OR  TITLE-ABS-KEY ( "dl-ketamine*" )  OR  TITLE-ABS-KEY ( "Etamine*" )  OR  TITLE-ABS-KEY ( "Esketamine*" )  OR  TITLE-ABS-KEY ( "Ivanes*" )  OR  TITLE-ABS-KEY ( "kalipsol*" )  OR  TITLE-ABS-KEY ( "Kanox*" )  OR  TITLE-ABS-KEY ( "Keiran*" )  OR  TITLE-ABS-KEY ( "Keta" )  OR  TITLE-ABS-KEY ( "Keta-Hameln" )  OR  TITLE-ABS-KEY ( "Ketacor*" )  OR  TITLE-ABS-KEY ( "ketaject*" )  OR  TITLE-ABS-KEY ( "ketalar*" )  OR  TITLE-ABS-KEY ( "Ketalin*" )  OR  TITLE-ABS-KEY ( "Ketamax*" )  OR  TITLE-ABS-KEY ( "ketamine*" )  OR  TITLE-ABS-KEY ( "ketanest*" )  OR  TITLE-ABS-KEY ( "ketaset*" )  OR  TITLE-ABS-KEY ( "Ketava*" )  OR  TITLE-ABS-KEY ( "Ketaved*" )  OR  TITLE-ABS-KEY ( "Ketazol*" )  OR  TITLE-ABS-KEY ( "ketoject*" )  OR  TITLE-ABS-KEY ( "Ketolar*" )  OR  TITLE-ABS-KEY ( "Narkamon*" )  OR  ( "N-methyl-D-aspartate"  W/3  "receptor inhibit*" )  OR  ( "NMDA"  W/2  "receptor inhibit*" )  OR  TITLE-ABS-KEY ( "NMDA-antagonis*" )  OR  TITLE-ABS-KEY ( "Quetanex*" )  OR  TITLE-ABS-KEY ( "S-ketamine*" )  OR  TITLE-ABS-KEY ( "Spravato*" )  OR  TITLE-ABS-KEY ( "Velonarcon*" )  OR  TITLE-ABS-KEY ( "Vetalar*" ) ) ) )  AND NOT  ( INDEX ( medline ) ) )  AND  ( ORIG-LOAD-DATE  >  20201101 )  AND  ( LIMIT-TO ( SRCTYPE ,  "j" ) )  AND  ( EXCLUDE ( DOCTYPE ,  "ch" )  OR  EXCLUDE ( DOCTYPE ,  "bk" ) )  AND  ( LIMIT-TO ( SUBJAREA ,  "MEDI" )  OR  LIMIT-TO ( SUBJAREA ,  "NEUR" )  OR  LIMIT-TO ( SUBJAREA ,  "PHAR" )  OR  LIMIT-TO ( SUBJAREA ,  "NURS" )  OR  LIMIT-TO ( SUBJAREA ,  "BIOC" )  OR  LIMIT-TO ( SUBJAREA ,  "DENT" )  OR  LIMIT-TO ( SUBJAREA ,  "HEAL" )  OR  LIMIT-TO ( SUBJAREA ,  "MULT" ) )  AND  ( LIMIT-TO ( EXACTKEYWORD ,  "Human" )  OR  LIMIT-TO ( EXACTKEYWORD ,  "Humans" )  OR  EXCLUDE ( EXACTKEYWORD ,  "Nonhuman" ) )

#

# Web of Science

Ketamine Headache Facial Pain

Data updated 2022-09-25

**Web of Science Core Collection: Citation Indexes**

A&HCI , BKCI-SSH , BKCI-S , ESCI , CPCI-SSH , CPCI-S , SCI-EXPANDED , SSCI

Web of Science Core Collection

A&HCI , BKCI-SSH , BKCI-S , ESCI , CPCI-SSH , CPCI-S , SCI-EXPANDED , SSCI

[76](https://www-webofscience-com.myaccess.library.utoronto.ca/wos/woscc/summary/0e072dec-dc04-46a5-a499-7c4fe8a013f5-51b29dd4/date-descending/1)

**#2 AND #1** and **2020** or **2021** or **2022** (Publication Years)

**TS=("Bell Palsy" OR "Eye Pain" OR "Facial Hemiatroph*" OR "Facial Neuralgia" OR "Facial Nerve Diseas*" OR "Facial Nerve Injuries" OR "Facial Neuralgia" OR "Facial Pain" OR "Facial Paralysis" OR Headache OR "Headache Disorders" OR "Hemifacial Spasm" OR "Herpes Zoster Oticus" OR "Lingual Nerve Injuries" OR "Melkersson-Rosenthal Syndrome" OR "Mobius Syndrome" OR "Myofascial Pain Syndrome*" OR "Neck Pain" OR "Temporomandibular Joint Dysfunction Syndrome" OR "Trigeminal Nerve Diseases" OR "Trigeminal Nerve Injuries" OR "Trigeminal Neuralgia" OR "headache and facial pain" OR "facial nerve injury" OR "facial nerve paralysis" OR "hemifacial atrophy" OR "hemifacial spasm" OR "herpes zoster oticus" OR "melkersson rosenthal syndrome" OR "moebius syndrome" OR "trigeminal autonomic cephalalgia" OR "trigeminus neuralgia" OR (bell* NEAR/2 pals*) OR ("cranial facial*" NEAR/2 pain*) OR (craniofacial* NEAR/2 pain*) OR (cranio-facial* NEAR/2 pain*) OR (facial* NEAR/2 neuralg*) OR (facial* NEAR/2 pain*) OR (hemifacial NEAR/2 spasm*) OR (myofascia* NEAR/2 pain*) OR (orofacial* NEAR/2 neuralg*) OR (oro-facial* NEAR/2 neuralg*) OR (orofacial* NEAR/2 pain*) OR (oro-facial* NEAR/2 pain*) OR ("temporomandibular joint*" NEAR/2 d*sfunct*) OR ("temporo-mandibular joint*" NEAR/2 d*sfunct*) OR (TMJ NEAR/2 d*sfunct*) OR (trigemina* NEAR/2 nerv*) OR (trigemina* NEAR/2 neuralgi*) OR "7th cranial nerve injur*" OR "7th cranial nerve paraly*" OR "central facial nerve pals*" OR cephalalgi* OR cephalgi* OR earache* OR ear-ache* OR "face pain*" OR "facial diplegi*" OR "facial nerve injur*" OR "facial nerve pals*" OR "facial nerve paralys*" OR "facial nerve pares" OR "facial nerve trauma*" OR "facial neuralgi*" OR "facial pain*" OR "facial pals*" OR "facial paralys*" OR "facial pares" OR facialgia* OR "facialis paralys" OR glossalgi* OR headache* OR head-ache* OR heavy-headedness OR "hemicrania continua" OR "hemifacial atroph*" OR "hemifacial spas*" OR "herpes zoster oticus" OR "melkersson rosenthal syndrom*" OR migrain* OR "moebius syndrome*" OR ophthalmoplegi* OR prosopoplegia* OR "seventh cranial nerve injur*" OR "seventh cranial nerve paralys*" OR "slit ventricle syndrome*" OR SUNA OR SUNCT OR "temporal arteritis" OR toothache* OR tooth-ache* OR "trigeminus neuralgi*")**

[193,629](https://www-webofscience-com.myaccess.library.utoronto.ca/wos/woscc/summary/f455c979-f2d4-43f8-8bb4-28d8fdcc0b2b-51b2966d/date-descending/1)

**TS=(Ketamine OR 690G0D6V8H OR "6740-88-1" OR Anesject* OR Brevinaze* OR Cyclohexanone* OR "2-(2-chlorophenyl)-2-(methylamino)cyclohexanone" OR "CLSTA 20" OR "ci 581" OR ci581 OR calipsol* OR calypsol* OR dl-ketamine* OR Etamine* OR Esketamine* OR Ivanes* OR kalipsol* OR Kanox* OR Keiran* OR Keta OR Keta-Hamel* OR Ketacor* OR ketaject* OR ketalar* OR Ketalin* OR Ketamax* OR ketamine* OR ketanest* OR ketaset* OR Ketava* OR Ketaved* OR Ketazol* OR ketoject* OR Ketolar* OR Narkamon* OR (N-methyl-D-aspartate NEAR/3 "receptor inhibit*") OR (NMDA NEAR/2 "receptor inhibit*") OR NMDA-antagonis* OR Quetanex* OR S-ketamine* OR Spravato* OR Velonarcon* OR Vetalar*)**

[44,099](https://www-webofscience-com.myaccess.library.utoronto.ca/wos/woscc/summary/d112b435-d841-4403-970d-9b4fd95f2de8-51b2966f/date-descending/1)

#

# ClinicalTrials.Gov


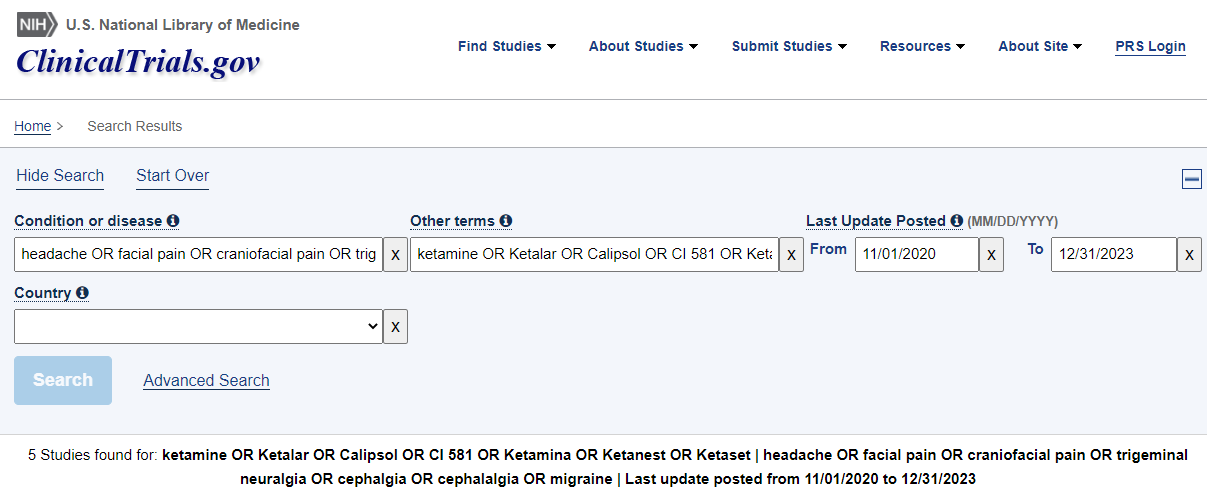


# [Search of: ketamine OR Ketalar OR Calipsol OR CI 581 OR Ketamina OR Ketanest OR Ketaset | headache OR facial pain OR craniofacial pain OR trigeminal neuralgia OR cephalgia OR cephalalgia OR migraine | Last update posted from 11/01/2020 to 12/31/2023 - List Results - ClinicalTrials.gov](https://clinicaltrials.gov/ct2/results?cond=headache+OR+facial+pain+OR+craniofacial+pain+OR+trigeminal+neuralgia+OR+cephalgia+OR+cephalalgia+OR+migraine&term=ketamine+OR+Ketalar+OR+Calipsol+OR+CI+581+OR+Ketamina+OR+Ketanest+OR+Ketaset&lupd_s=11%2F01%2F2020&lupd_e=12%2F31%2F2023&cntry=&state=&city=&dist=&Search=Search)

# 5 Studies found for: ketamine OR Ketalar OR Calipsol OR CI 581 OR Ketamina OR Ketanest OR Ketaset | headache OR facial pain OR craniofacial pain OR trigeminal neuralgia OR cephalgia OR cephalalgia OR migraine | Last update posted from 11/01/2020 to 12/31/2023

# WHO ICTRP


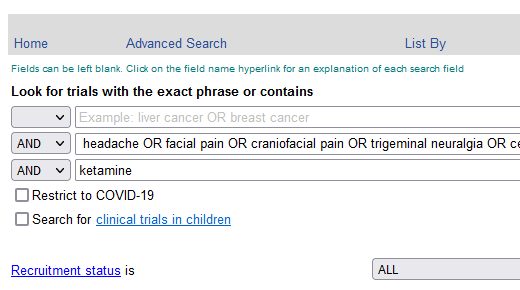


Synonyms

- PAIN, CRANIOFACIAL, craniofacial pain - FACE ACHE, FACE ACHE (FINDING), FACE PAIN, FACE; PAIN, FACIAL; PAIN, HEADACHE, PAIN FACE, PAIN IN FACE, PAIN IN FACE (FINDING), PAIN, FACE, PAIN, FACIAL, PAIN; FACE, PAIN; FACIAL, PAIN;FACE, facial pain - FORTIFICATION SPECTRA, HEADACHE MIGRAINOUS, MIGRAINOUS HEADACHE, MIGRAINOUS NEURALGIA, migraine - ABDOMEN PAIN, CEPHALALGIA, CEPHALALGIAS, CEPHALGIA, CEPHALGIAS, CEPHALODYNIA, CEPHALODYNIAS, CRANIAL PAIN, CRANIAL PAINS, FACIAL PAIN, FACIAL PAIN NOS, HEAD PAIN, HEAD PAINS, HEAD; PAIN, PAIN HEAD, PAIN IN HEAD NOS, PAIN, CRANIAL, PAIN, HEAD, PAIN; HEAD, PAIN;HEAD, PAINS, CRANIAL, PAINS, HEAD, headache - 2-(2-CHLOROPHENYL)-2-(METHYLAMINO)CYCLOHEXANONE, ketamine


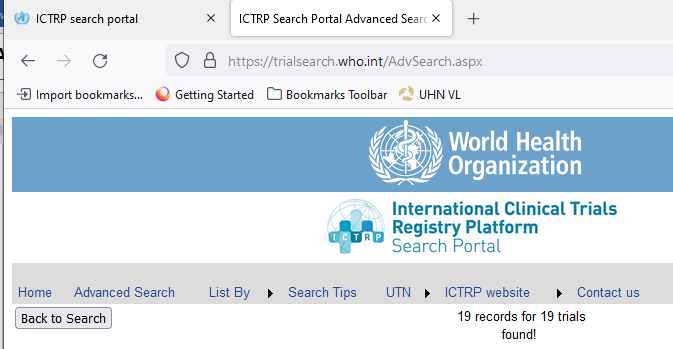


19 clinical trials are included in the RIS results files.

| **Recruitment status** | **Prospective Registration** | **Main ID** |  | **Public Title** | **Date of Registration** | **Results available** |  |
| --- | --- | --- | --- | --- | --- | --- | --- |
|  | Not recruiting | Yes | NCT05306899 |  | [Multi-center RCT of IV Ketamine Efficacy and Safety in Chronic Daily Headaches](https://trialsearch.who.int/Trial2.aspx?TrialID=NCT05306899) | 2022-03-02 |  |
|  | Recruiting | Yes | NCT04814381 |  | [Ketamine + Magnesium for Chronic Cluster Headache (KETALGIA)](https://trialsearch.who.int/Trial2.aspx?TrialID=NCT04814381) | 2021-03-18 |  |
|  | Recruiting | Yes | IRCT20120215009014N387 |  | [Effect of low dose intravenous ketamine versus placebo on the prevention of headache after spinal anesthesia in patients who are candidates for cesarean section](https://trialsearch.who.int/Trial2.aspx?TrialID=IRCT20120215009014N387) | 2021-03-09 |  |
|  | Not Recruiting | Yes | TCTR20200409009 |  | [Accuracy of Clinical Prediction Score for Serious Intracranial Causes of Acute Non-Traumatic Headache in Emergency Department](https://trialsearch.who.int/Trial2.aspx?TrialID=TCTR20200409009) | 2020-04-09 |  |
|  | Not Recruiting | No | IRCT20170224032753N2 |  | ["Comparison the Effect of Intravenous Ketamine with Standard Treatment in Migraine Patients"](https://trialsearch.who.int/Trial2.aspx?TrialID=IRCT20170224032753N2) | 2020-03-22 |  |
|  | Not recruiting | Yes | NCT04179266 |  | [Effect of Ketamine Intranasal Spray in Treatment of Chronic Cluster Headache](https://trialsearch.who.int/Trial2.aspx?TrialID=NCT04179266) | 2019-11-25 |  |
|  | Recruiting | No | IRCT20180108038276N3 |  | [Comparison of intranasal ketamine and intravenous ketorolac in reducing non-traumatic acute headache](https://trialsearch.who.int/Trial2.aspx?TrialID=IRCT20180108038276N3) | 2019-09-29 |  |
|  | Not Recruiting | Yes | EUCTR2019-001260-29-DK | 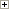 | [Clinical pilot study for evaluation of the effect of ketamine intranasal spray in treatment of chronic Cluster Headache](https://trialsearch.who.int/Trial2.aspx?TrialID=EUCTR2019-001260-29-DK) | 2019-06-12 |  |
|  | Not recruiting | Yes | NCT03896256 |  | [Ketamine for Refractory Chronic Migraine: a Pilot Study](https://trialsearch.who.int/Trial2.aspx?TrialID=NCT03896256) | 2019-03-26 |  |
|  | Recruiting | Yes | IRCT20190202042589N1 |  | [The Effect of Low-Dose Intravenous Ketamine on prevention of post dural puncture headache](https://trialsearch.who.int/Trial2.aspx?TrialID=IRCT20190202042589N1) | 2019-02-14 |  |
|  | Not recruiting | No | NCT03221569 |  | [Ketamine v. Ketorolac for Management of Generalized Tension Type Headache](https://trialsearch.who.int/Trial2.aspx?TrialID=NCT03221569) | 2017-07-17 |  |
|  | Recruiting | Yes | NTR6480 |  | [S-ketamine for acute and chronic headache after brainsurgery](https://trialsearch.who.int/Trial2.aspx?TrialID=NTR6480) | 2017-07-04 |  |
|  | Not recruiting | Yes | NCT03152955 |  | [Postoperative Analgesia in Patients With Microvascular Decompression](https://trialsearch.who.int/Trial2.aspx?TrialID=NCT03152955) | 2017-05-04 |  |
|  | Not recruiting | No | NCT03081416 |  | ["THINK Trial: Treatment of Headache With IntraNasal Ketamine: A Randomized Controlled Trial Evaluating the Efficacy of Intranasal Ketamine Versus Standard Therapy in the Management of Primary Headache Syndromes in the Emergency Department"](https://trialsearch.who.int/Trial2.aspx?TrialID=NCT03081416) | 2017-03-10 |  |
|  | Not recruiting | Yes | NCT02735343 |  | [The CHECK Trial: A Comparison of Headache Treatment in the Emergency Department: Compazine Versus Ketamine](https://trialsearch.who.int/Trial2.aspx?TrialID=NCT02735343) | 2016-03-31 |  |
|  | Not recruiting | Yes | NCT02697071 |  | [Ketamine for Acute Migraine in the Emergency Department](https://trialsearch.who.int/Trial2.aspx?TrialID=NCT02697071) | 2016-02-24 |  |
|  | Not recruiting | Yes | NCT02657031 |  | [The CHECK Trial: A Comparison of Headache Treatment in the ED: Compazine Versus Ketamine](https://trialsearch.who.int/Trial2.aspx?TrialID=NCT02657031) | 2016-01-13 | Yes |
|  | Not recruiting | Yes | NCT02403687 |  | [Prospective Analgesic Compound Efficacy (PACE) Study](https://trialsearch.who.int/Trial2.aspx?TrialID=NCT02403687) | 2015-03-05 |  |
|  | Not Recruiting | No | ISRCTN18446134 |  | [Comparative study of the efficacy and safety of intranasal ketamine and midazolam for the acute treatment of migraine with prolonged aura](https://trialsearch.who.int/Trial2.aspx?TrialID=ISRCTN18446134) | 2003-09-12 |  |
|  |  |  |  |  |  |  |  |
